# Asynchronous Responses of Plants, Soils, and Microbes to Snow Cover Change Across Terrestrial Ecosystems: A Global Meta-Analysis

**DOI:** 10.3390/plants14203172

**Published:** 2025-10-15

**Authors:** Yafei Shi, Yuning Zhang, Xing Hong, Xiaoni Liu

**Affiliations:** Pratacultural College, Gansu Agricultural University, Lanzhou 730070, China; shiyf@gsau.edu.cn (Y.S.); 1073324020042@st.gsau.edu.cn (Y.Z.); 20230202026@st.gsau.edu.cn (X.H.)

**Keywords:** snow cover, plant–soil interactions, microbial response, ecosystem coupling, meta-analysis

## Abstract

Snow cover, as a critical component of the global climate system, strongly influences ecological processes in cold and temperate regions. However, how different ecosystem components—plants, soils, and microbes—respond to snow cover change remains poorly understood, especially in terms of their coordination. Here, we conducted a global meta-analysis of 1986 single and 1047 paired observations from snow manipulation experiments across diverse terrestrial ecosystems. Our results showed that snow removal generally reduced SWC and microbial diversity, whereas snow addition exerted smaller or more variable influences across ecosystem components. Among all variables, the effect of snow cover change on soil water content was most pronounced, whereas its impacts on other factors were generally weak. Notably, the direction and magnitude of responses often differed among ecosystem components exposed to the same treatments. Pairwise comparisons revealed frequent mismatches between plant and soil organism responses, indicating substantial ecosystem-level decoupling across biomes. These findings support the ecosystem asynchrony hypothesis and highlight the need for integrated approaches that link aboveground and belowground processes. Our study improves the understanding of ecosystem stability under changing snow regimes and provides insights for predicting future terrestrial responses to global climate change.

## 1. Introduction

Terrestrial ecosystems are profoundly influenced by changes in snow cover, including both snow accumulation and reduction, which are key components of ongoing global environmental change [1]. Snow, as a crucial component of the hydrological cycle and a key indicator of climate change, exerts significant effects on plants, soils, and microorganisms [2]. Snow cover typically provides essential insulation and thermal buffering and can serve as an important water source for plant growth in the subsequent growing season. However, due to climate warming and other contributing factors, surface temperatures have been steadily rising in recent years, leading to a notable decline in both the extent and duration of snow cover, notably across the Northern Hemisphere. Therefore, understanding how snow influences different components of terrestrial ecosystems is critical in the context of global environmental change.

Changes in snow cover affect various ecological components through alterations in microclimate and soil processes. Previous studies have shown that winter snow cover strongly influences the spatial distribution of soil moisture and soil temperature, thereby modifying soil environmental conditions and indirectly shaping microbial activity—especially under conditions of increased snow accumulation [3,4,5]. For example, greater snowpack acts as an effective insulator between soil and air, elevating surface temperatures and enhancing soil thermal protection, which may in turn promote plant growth in the following season [6]. Moreover, during snowmelt periods, sustained water input can significantly influence soil physicochemical properties [7,8,9]. Warmer soils may also stimulate microbial metabolism, thereby potentially facilitating sustained nitrogen fixation [10]. However, research by Wang et al., 2024 [11] suggests that responses to snow cover changes may vary across different hierarchical levels within ecosystems. A variety of ecological hypotheses and conceptual frameworks have been proposed to explain the differential responses of ecosystem components [12]. Among them, the Asynchrony Hypothesis, posits that distinct ecosystem compartments—such as vegetation, soil microbes, and nutrient pools—respond in a temporally decoupled manner to shared external stressors, including elevated temperatures and reduced snow cover [13].

Over the past several decades, numerous snow manipulation experiments have been conducted worldwide to simulate and better predict the impacts of global change on terrestrial ecosystems [14]. A recent meta-analysis demonstrated that snow cover change alters soil microclimate and microbial processes, ultimately affecting vegetation productivity [15]. Although multiple meta-analyses have explored how snow cover change, under the context of global change, influences soil properties and processes [16,17], few have explicitly focused on the interactions across the plant–soil–microbe continuum, whose coupled functioning regulates fundamental ecosystem processes. Existing evidence further suggests that the effect of snow cover change on soil water content (SWC) tends to be the most pronounced, whereas its impacts on other factors appear generally weaker. Coupling in ecosystems refers to the synchronous and functionally linked responses of biotic compartments to a shared driver, whereas decoupling implies divergent or asynchronous dynamics. In other words, above- and belowground biota may exhibit distinct responses to snow cover change, which could lead to altered interactions and functional dynamics. This represents a major knowledge gap in understanding whether the current balance of linkages among plants, soils, and microorganisms will shift under future environmental changes. Such a gap limits our ability to predict how snow cover change may affect ecosystem structure and function in a globally changing environment. Therefore, it is imperative to integrate plant, soil, and microbial responses to snow cover change to evaluate their contributions to ecosystem-level coupling or decoupling between above- and belowground compartments.

In this study, we conducted a global meta-analysis of snow manipulation experiments across terrestrial ecosystems to quantify the responses of aboveground vegetation (e.g., biomass, ANPP, and community density), soil physicochemical properties (e.g., total nitrogen, pH, soil moisture, and temperature), and microbial attributes (e.g., microbial biomass carbon and nitrogen) to snow cover change. Additionally, we analyzed paired data linking plant traits, soil conditions, and microbial biomass to explore the degree of coordination or decoupling in their responses. Specifically, we addressed the following questions: (1) How does snow cover change affect the structure and functioning of plants, soils, and microbes individually? (2) To what extent do these components exhibit similar or divergent response patterns across treatments? (3) Do these patterns reflect coupled or decoupled ecosystem responses to snow cover change?

## 2. Results

### 2.1. Overall Responses of Plants, Soils, and Microbes to Snow Cover Change

**Figure 1:**
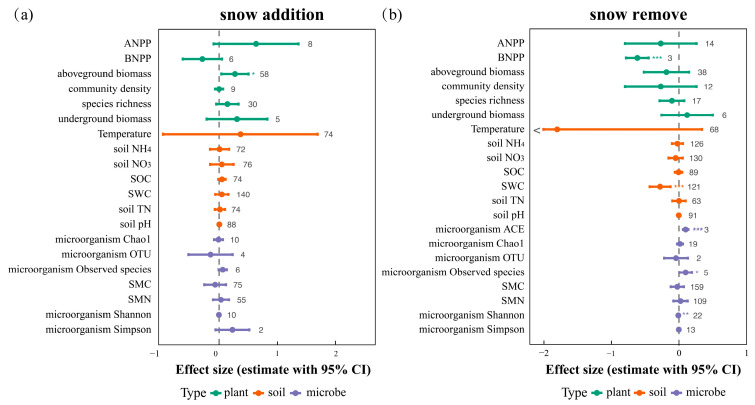
Effects of snow cover change on plants, soils, and microorganisms under snow addition (**a**) and snow reduction (**b**). Points represent mean effect sizes (estimate) with 95% confidence intervals. *, **, and *** indicate significance at *p* < 0.05, 0.01, and 0.001 levels, respectively. Sample sizes (n) are displayed adjacent to each variable. Abbreviations: ANPP—Aboveground net primary productivity; BNPP—Belowground net primary productivity; Aboveground biomass—Biomass above the soil surface; Community density—Density of individuals in the plant community; Species richness—Number of species observed in a community; Underground biomass—Biomass below the soil surface; Temperature—Soil temperature; Soil NH_4_^+^—Soil ammonium nitrogen; Soil NO_3_^−^—Soil nitrate nitrogen; SOC—Soil organic carbon; SWC—Soil water content; Soil pH—Soil acidity/alkalinity; Microorganism Chao1—Microbial Chao1 richness estimator; Microorganism OTU—Microbial operational taxonomic units; Microorganism Observed species—Number of observed microbial species; MBC—Microbial biomass carbon; MBN—Microbial biomass nitrogen; Microorganism Shannon—Microbial Shannon diversity index; Microorganism Simpson—Microbial Simpson diversity index.

Across all experiments globally, snow addition did not significantly affect most ecosystem components, except for a moderate increase trend in aboveground biomass [effect size (hereafter abbreviated as es) = 0.258, 95% confidence interval (hereafter abbreviated as CI) = −0.038 to 0.479; Figure 1a]. However, this response exhibited high heterogeneity (Q_t_ = 3378.224, *df* = 57, *p* < 0.001). In contrast, snow reduction elicited pronounced effects across belowground components (Figure 1b). Specifically, it significantly reduced belowground net primary productivity (BNPP; es = −0.616, 95% CI = −0.783 to −0.449), soil water content (SWC; es = −0.279, 95% CI = −0.433 to −0.125), and microbial diversity metrics, including the ACE index (es = 0.099, 95% CI = 0.059 to 0.139), species richness (es = 0.098, 95% CI = 0.005 to 0.191), and the Shannon index (es = −0.009, 95% CI = −0.017 to −0.003) (see Tables S1 and S2 in the Supporting Information).

Notably, the effect of snow reduction on SWC showed substantial heterogeneity (Q_t_ = 7306.953, *df* = 120, *p* < 0.001), indicating considerable variation in treatment outcomes. Although the effect of snow cover change on soil temperature was not statistically significant, its high variability suggests potential ecological sensitivity to snow regime shifts across experimental contexts.

### 2.2. Responses to Snow Cover Change Across Different Ecosystem Types

**Figure 2:**
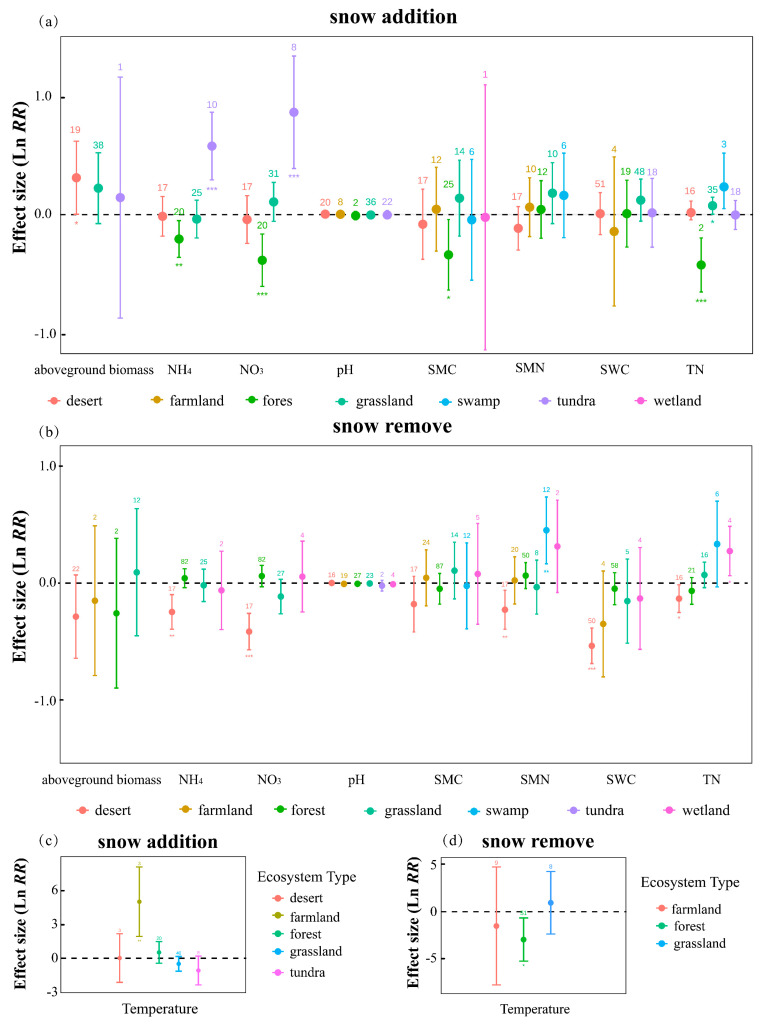
Effects of snow cover change on ecosystem variables under (**a**) snow addition and (**b**) snow removal, and their relationships with mean annual temperature under (**c**) snow addition treatment and (**d**) snow removal treatment. Values are mean ± 95% CI of Ln RR; numbers denote sample sizes; *, **, *** indicate significance at *p* < 0.05, 0.01, and 0.001, respectively.

To evaluate the responses of different ecosystem types to snow cover change, we analyzed treatment effects within each biome. Under snow addition (Figure 2a), deserts showed the strongest increase in aboveground biomass (es = 0.312, 95% CI = 0.003 to 0.621), while farmlands (es = 4.984, 95% CI = 1.927 to 8.041) exhibited substantial warming. In contrast, forests experienced significant declines in soil ammonium, nitrate, and moisture, indicating sensitivity to snow accumulation. Grasslands showed a mild but significant increase in total nitrogen (es = 0.075, 95% CI = 0.003 to 0.147). Under snow removal (Figure 2b), deserts exhibited consistent negative responses across multiple soil variables, and forests showed pronounced declines in soil temperature (es = −3.006, 95% CI = −5.298 to −0.714), highlighting their vulnerability to snow loss. Temperature was the most responsive variable and was analyzed separately. Farmland showed a marked increase in soil temperature (es = 4.983, 95% CI = 1.926 to 8.040) (Figure 2c), and forests also showed substantial reductions in soil temperature (es = −3.006, 95% CI = −5.298 to −0.714) (Figure 2d), highlighting their vulnerability to snow loss. Given the relatively limited data for swamp (*n* = 12) and wetland (*n* = 4) ecosystems regarding microbial and total nitrogen, the corresponding results are presented for illustrative purposes only. Overall, these results highlight strong heterogeneity in snow responses across biomes, with deserts and forests being particularly sensitive.

### 2.3. Responses Across Different Snow Manipulation Methods

**Figure 3:**
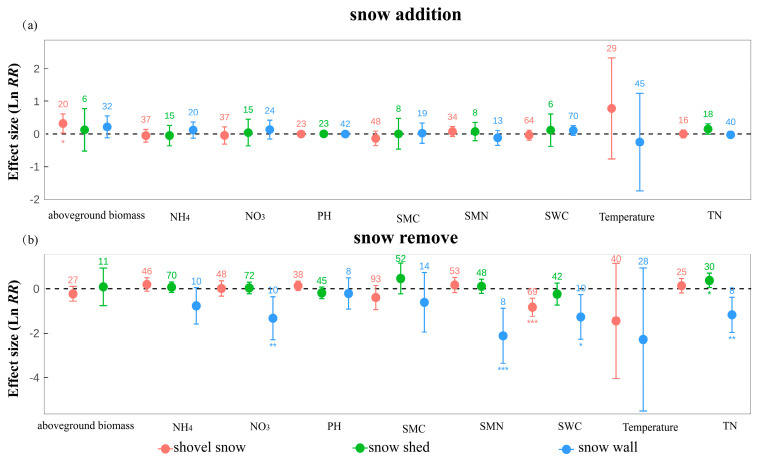
Effects of snow cover change across three manipulation methods on ecosystem variables under (**a**) snow addition and (**b**) snow removal. Values are Ln RR ± 95% CI; numbers indicate sample sizes; *, **, *** denote *p* < 0.05, 0.01, 0.001.

Across the three modes of snow manipulation, under snow addition, only the shovel snow treatment led to a statistically significant increase in aboveground biomass (es = 0.322, 95% CI = 0.026 to 0.617; Figure 3). No significant effects were detected under either snow shed or snow wall treatments across measured variables. Under snow removal, shovel snow resulted in a pronounced decline in soil water content (es = −0.831, 95% CI = −1.241 to −0.422), while snow shed treatments significantly increased total nitrogen (es = 0.376, 95% CI = 0.050 to 0.701). Although snow wall manipulations yielded significant reductions in soil nitrate, soil water content, total nitrogen, and microbial biomass nitrogen, these findings are based on limited sample sizes (*n* = 8–10). Different snow addition and removal strategies may lead to variable outcomes, but these effects warrant further investigation within the context of specific experimental settings.

### 2.4. Depth-Dependent Soil and Microbial Responses

**Figure 4:**
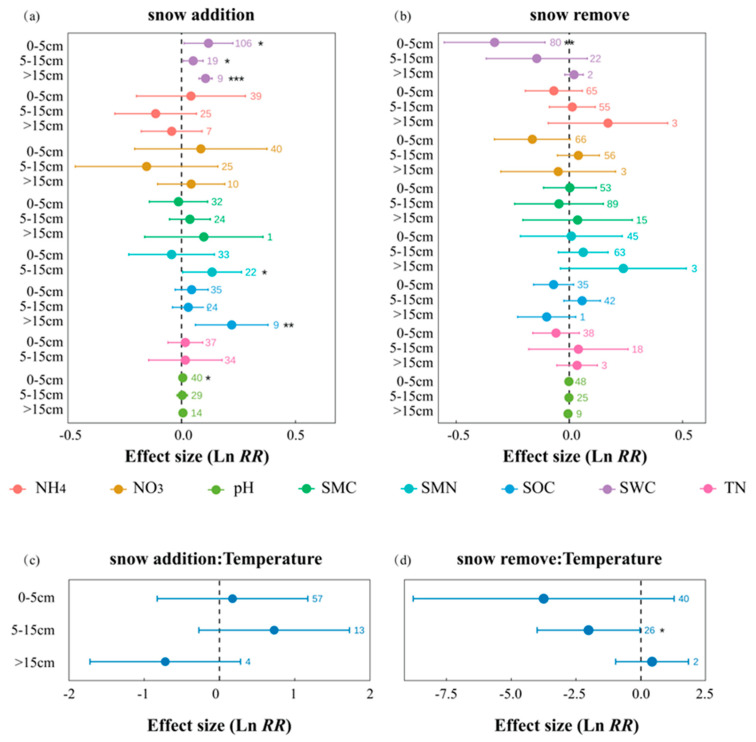
Effects of snow cover change on soil variables at different depths: (**a**) snow addition and (**b**) snow removal; (**c**,**d**) relationships with mean annual temperature. Values are ln RR ± 95% CI; numbers indicate sample sizes. Variables: NH_4_, NO_3_, pH, SMC, SMN, SOC, SWC, TN. *, **, *** denote *p* < 0.05, 0.01, 0.001.

At different soil depths, under snow addition (Figure 4a), significant increases were observed in SMN at 5–15 cm (es = 0.134, 95% CI = 0.004 to 0.263), and in SWC at both 0–5 cm (es = 0.118, 95% CI = 0.011 to 0.225) and 5–15 cm (es = 0.105, 95% CI = 0.076 to 0.134). Although snow addition also tended to increase SOC and SWC in deeper layers (>15 cm), limited sample sizes (e.g., n = 9) weaken the statistical reliability of these effects. In contrast, snow removal (Figure 4b) significantly reduced SWC at 0–5 cm (es = −0.327, 95% CI = −0.548 to −0.106) and soil temperature at 5–15 cm (es = −2.022, 95% CI = −4.008 to −0.036). Notably, soil temperature exhibited wide confidence intervals across all depths, indicating high variability and sensitivity to snow regime changes (Figure 4c,d). In general, these results indicate that shallow soil layers are more responsive to snow cover change, particularly in terms of moisture and thermal dynamics (see Tables S3 and S4 in the Supporting Information).

### 2.5. Plant–Soil Correlations Under Snow Cover Change

**Figure 5:**
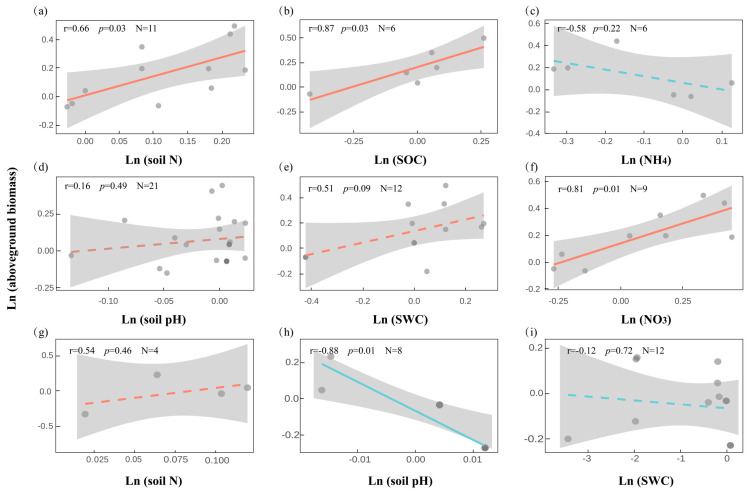
Relationships between aboveground biomass and key soil physicochemical variables in response to snow cover change ((**a**–**f**): snow addition; (**g**–**i**): snow removal).

We analyzed the correlations between aboveground vegetation and soil physicochemical responses to snow cover change based on paired experimental results from the same study. Under snow addition (Figure 5a–f), aboveground biomass showed significant positive correlations with TN (r = 0.66, *p* = 0.028), SOC (r = 0.87, *p* = 0.025), and soil nitrate (NO_3_^−^) (r = 0.81, *p* = 0.009), suggesting potential nutrient-mediated coupling between plant and soil responses. In contrast, associations with soil ammonium (NH_4_^+^) (r = −0.58, *p* = 0.223), SWC (r = 0.51, *p* = 0.089), and pH (r = 0.16, *p* = 0.489) were non-significant. Under snow removal (Figure 5g–i), only soil pH was significantly correlated with aboveground biomass (r = −0.88, *p* = 0.004), while other variables such as TN (r = 0.54, *p* = 0.459) and SWC (r = −0.12, *p* = 0.715) showed weak or nonsignificant relationships. The lack of consistent correlations suggests potential decoupling between plant productivity and soil processes under snow loss scenarios, possibly due to increased environmental stress and functional asymmetry.

### 2.6. Microbe–Soil Correlations Under Snow Cover Change

**Figure 6:**
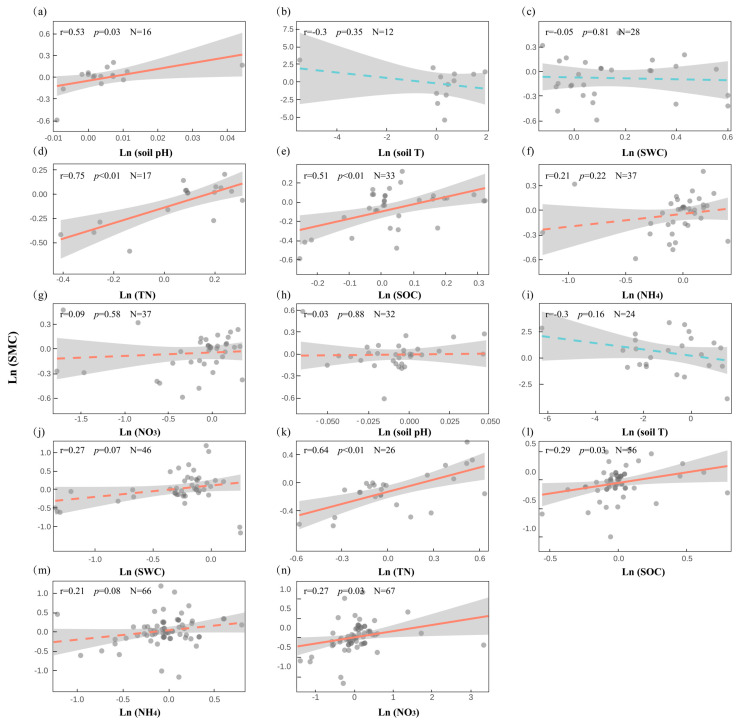
Relationships between microbial biomass carbon (MBC) and key soil environmental variables under snow cover change ((**a**–**g**): snow addition; (**h**–**n**): snow removal).

To explore how snow cover change affects microbial communities, we analyzed pairwise correlations between MBC, MBN, and key soil physicochemical properties, using matched data derived from the same studies. In the case of MBC and soil variables, we found that under snow addition (Figure 6a–g), MBC was positively correlated with pH (r = 0.53, *p* = 0.034), SOC (r = 0.51, *p* = 0.003), and TN (r = 0.75, *p* = 0.001), suggesting that nutrient enrichment and pH buffering promote microbial carbon accumulation. Other variables, including temperature and moisture, showed no significant relationships. Under snow removal (Figure 6h–n), MBC remained significantly associated with SOC (r = 0.29, *p* = 0.032), TN (r = 0.64, *p* < 0.001), and nitrate (r = 0.27, *p* = 0.026), while correlations with SWC (r = 0.27, *p* = 0.071) and ammonium (r = 0.21, *p* = 0.083) were marginal. pH (r = 0.03, *p* = 0.877) and temperature (r = −0.30, *p* = 0.157) exhibited weak or negligible effects. Overall, microbial carbon dynamics appear tightly linked to nutrient availability, particularly SOC and TN, but are less responsive to hydrological and thermal factors under snow disturbance.

**Figure 7:**
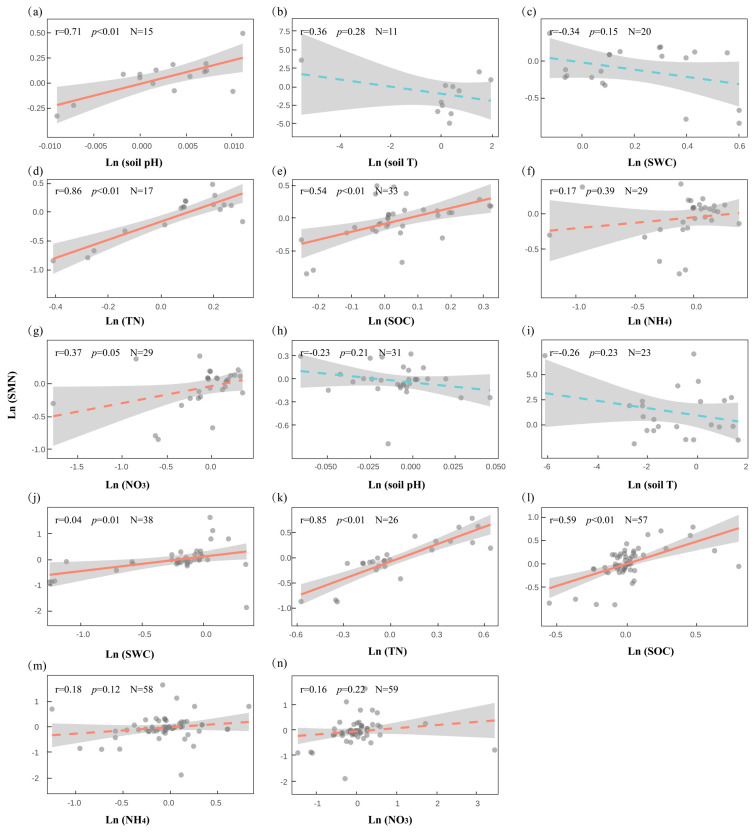
Relationships between microbial biomass nitrogen (SMN) and key soil environmental variables under snow cover change ((**a**–**g**): snow addition; (**h**–**n**): snow removal).

In the case of MBN and soil variables, we found that, under snow addition (Figure 7a–g), SMN was significantly positively correlated with soil pH (r = 0.71, *p* = 0.003), SOC (r = 0.54, *p* = 0.001), and TN (r = 0.86, *p* < 0.001), indicating enhanced microbial nitrogen accumulation under improved nutrient availability and buffered pH conditions. Under snow removal (Figure 7h–n), SMN also exhibited positive correlations with SWC (r = 0.40, *p* = 0.013), SOC (r = 0.59, *p* < 0.001), and TN (r = 0.85, *p* < 0.001), suggesting microbial nitrogen is sensitive to reductions in soil moisture and nutrient supply. Ammonium and nitrate did not show significant correlations under either treatment, while pH and temperature displayed weak and inconsistent relationships. These findings underscore the importance of nutrient-mediated pathways in shaping microbial resilience to changing snow regimes, suggesting that nutrient coupling plays a dominant role in regulating microbial carbon responses. Moreover, microbial nitrogen dynamics appear tightly linked to nutrient and moisture availability, but exhibit contrasting patterns under snow addition versus snow removal, highlighting differential regulatory mechanisms across snow manipulation strategies.

## 3. Discussion

### 3.1. Component-Specific Responses to Snow Cover Change

In this study, we found that plants, soils, and microbes showed clearly different responses to changes in snow cover. Overall, plants exhibited variable but not consistently stronger responses compared with soils and microbes, with greater variability in the mean effect sizes of related indicators. The consistent response directions across experiments suggest that vegetation productivity and community structure may be more susceptible to regulation by snow dynamics. These observations are consistent with previous findings [7,18], and likely reflect the rapid physiological adjustments plants make in response to shifts in temperature and soil moisture.

In contrast, responses of soil and microbial indicators were generally weaker, with mean effect sizes concentrated near zero (see Figure 1). Among all measured variables, soil water content showed the most pronounced response to snow cover change, while the responses of other factors remained generally weak. Snow reduction consistently led to decreased soil moisture. This underscores the likely crucial role of snowpack in regulating water input in arid and cold environments, and aligns with the conclusions drawn by Hui [7]. However, considerable variability in soil temperature responses, reflected by wide confidence intervals, suggests that these effects are context-dependent, likely driven by heterogeneity in experimental design and climatic conditions [19,20].

Compared to plant and soil variables, microbial indicators showed greater statistical significance. This may reflect a dual sensitivity to both temperature and resource constraints, as well as potential time-lagged physiological reactions, although direct mechanistic evidence is lacking [21,22]. This result highlights the pronounced heterogeneity in the effects of snow cover change across ecosystem components, underscoring the importance of integrating such differences into future modeling frameworks to help improve the ecological accuracy of predicted responses [23,24].

### 3.2. Drivers of Response Variability Across Ecosystems and Treatments

Ecosystem responses to snow cover change varied markedly across types, driven by differences in water and thermal dependence. In deserts, where snowmelt serves as a key water source, plants showed strong responses due to nutrient-sensitive organs and high water-use efficiency [20]. The prevalence of biocrusts and dominant water limitation further amplified these effects [25]. In contrast, forest ecosystems exhibited greater sensitivity to snow addition, particularly in root zone insulation [25], while soil and microbes responded weakly, buffered by stable microclimatic conditions. Overall, water-limited systems responded more strongly to snow reduction, whereas thermally regulated systems were more sensitive to snow addition.

Manipulation method played a key role in modulating component responses (Figure 4). Snow walls tended to exert the strongest impact on soil by altering snow accumulation, wind exposure, albedo, and evapotranspiration, creating frost-prone microclimates and likely intensifying soil desiccation and physicochemical shifts [20,26,27]. In contrast, shovel snow and snow shed treatments exerted milder effects, primarily by modifying snow persistence or depth. For microbes, these changes affected carbon input and temperature, limiting metabolic activity under reduced litter supply and lower soil temperatures [22]. Although microbial dormancy offers some buffering [24], intense disturbances can still alter community functions.

Soil depth also shaped response patterns, especially in the 0–5 cm layer (Figure 4), where direct exposure to atmospheric variation made temperature and moisture more dynamic [28,29]. With increasing depth, thermal inertia and water retention increased, dampening external signals and stabilizing microbial and soil responses [30]. Notably, under snow wall treatments, surface moisture declined significantly, while deeper layers remained relatively unaffected, indicating a “surface-sensitive, depth-buffered” pattern.

Because of the high heterogeneity observed in our results, we conducted a model-averaged analysis of predictor importance to better identify potential drivers of heterogeneity (e.g., ecosystem type, snow manipulation method, and climatic variables). Although we attempted to include climatic factors such as snow duration in the analysis, the available data coverage was only about 40%, which precluded drawing more reliable and comprehensive conclusions. Nevertheless, we ranked ecosystem type, snow manipulation method, and climatic variables by model-averaged importance (Supplementary Figures S5 and S6) and performed subgroup analyses based on ecosystem type and snow manipulation method—variables consistently reported across all studies—to better assess differences in responses to snow cover change among ecosystems and manipulation approaches.

These divergent responses reflect intrinsic differences in physical traits (e.g., heat capacity, permeability), physiological strategies (e.g., nutrient allocation, metabolic plasticity), and ecological adaptations (e.g., water-use efficiency, dormancy). Plants respond rapidly and directly, soils act as thermal and hydrological buffers, and microbes display delayed, constrained responses. This mechanistic may underpin the partial decoupling observed among ecosystem components and highlights the need for integrated, cross-scale models to predict system-level outcomes under changing snow regimes [31,32].

### 3.3. Partial Decoupling of Ecosystem Components

Ecosystem functioning relies on the coordination among plants, soils, and microbes. Previous studies suggest that snow cover change alters soil physicochemical properties, which in turn affect plant and microbial responses [33]. Based on 1047 paired observations linking soil variables with plant and microbial metrics, we found frequent mismatches in their responses to snow manipulation (Figure 1). For instance, under snow reduction, aboveground biomass declined significantly while microbial biomass remained unchanged or shifted in the opposite direction, indicating widespread partial decoupling among ecosystem components.

This pattern is consistent with the Hierarchical Response Framework [12] and Ecosystem Asynchrony Hypothesis [34], both of which emphasize differences in temporal sensitivity and regulatory pathways across components. Plants often respond rapidly to snow-induced thermal and moisture changes, while microbial responses are delayed, shaped by dependencies on plant-derived substrates and constrained by complex environmental interactions [33,35]. Functionally, such decoupling may reduce ecosystem resilience, potentially destabilizing key biogeochemical processes like carbon turnover, water retention, and nutrient cycling [36]. However, a certain degree of decoupling may reflect functional redundancy, helping sustain key ecosystem processes under complex or compounded disturbances [37,38]. Understanding how different components of terrestrial ecosystems respond to snow cover change—and the mechanisms underlying these responses—is critical for maintaining, restoring, and forecasting ecosystem functioning under global change. Isolated observations of single subsystems are insufficient to capture the integrated dynamics of the whole ecosystem. Future research must consider cross-component interactions within ecosystems to inform effective strategies for climate mitigation and adaptation. Notably, our correlation analyses (Figure 5, Figure 6 and Figure 7) highlight that soil characteristics play a central role in mediating plant and microbial responses. These findings suggest that snow cover itself may exert limited or transient effects, whereas soil properties appear to provide the primary pathways through which plants and microbes are affected.

### 3.4. Limitations and Future Directions

In addition to potential publication bias, meta-analysis may be influenced by other systematic biases such as language, cost, and familiarity biases [39,40]. Although we employed a comprehensive and systematic search strategy across multiple databases, more than half of the included studies originated from Eurasia, with a disproportionate concentration in temperate and cold-temperate biomes. This geographic imbalance reflects limited snowfall in tropical and arid regions, where snow manipulation is less ecologically relevant, but is unlikely to substantially compromise the broader applicability of our findings [41]. Moreover, most studies included only snow addition or removal treatments, with few simultaneously assessing plants, soils, and microbes under both conditions within a single experimental design.

Additional limitations arise from the exclusion of studies involving non-natural snow manipulations (e.g., mechanically generated snow) or those confounded by topographic heterogeneity such as slope-induced variation in snow depth. These design inconsistencies further highlight the importance of establishing standardized protocols for snow manipulation experiments that minimize confounding factors and enhance comparability across sites. Altogether, future snow cover research would benefit from prioritizing more geographically comprehensive, methodologically robust, and ecologically integrated approaches. By addressing current data and design limitations, these efforts are expected to enhance our predictive capacity for how terrestrial ecosystems respond to ongoing and future snow regime shifts.

## 4. Materials and Methods

### 4.1. Literature Search and Data Collection

**Figure 8:**
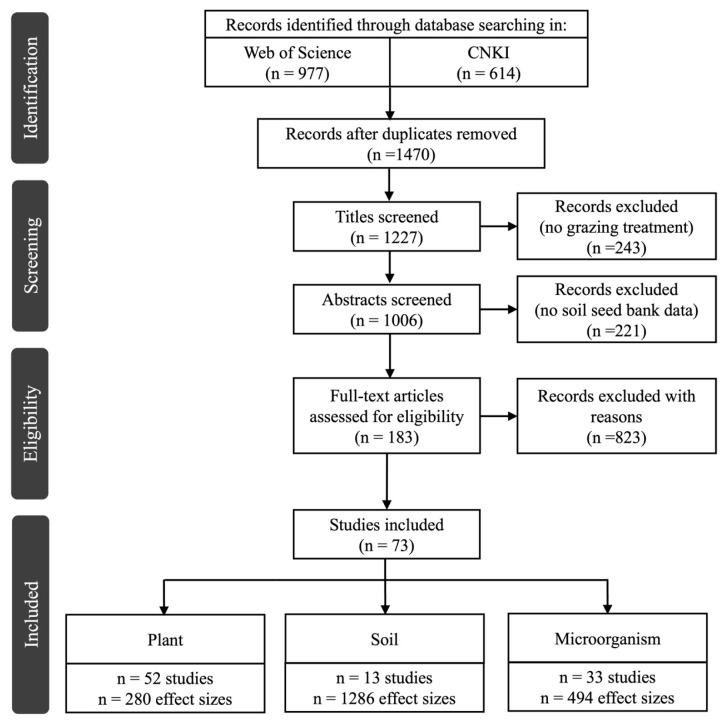
PRISMA (Preferred Reporting Items for Systematic reviews and Meta-Analyses) diagram representing the flow of information through a decision tree (i.e., the number of studies identified, rejected and accepted) (based on: www.prismastatement.org).

In January 2025, we conducted a comprehensive literature search using the Web of Science and China National Knowledge Infrastructure (CNKI) databases, following the Preferred Reporting Items for Systematic Reviews and Meta-Analyses (PRISMA) guidelines (Figure 8). The PRISMA checklist (Figure 8 and statement are provided in the Supplementary Materials.

The search terms included the following: snow OR snow cover AND plant OR plant phenology OR soil microorganism OR soil physical and chemical OR soil. Relevant studies were identified by screening the title, abstract, full text, and Supplementary Materials. The following criteria were applied to select eligible studies:

(1) Only studies in which snow cover manipulation was the sole experimental treatment were included. Those incorporating additional treatments (e.g., nutrient addition, warming, or irrigation) were excluded to avoid confounding effects.

(2) Studies involving manipulations that artificially advanced or extended the growing season were excluded, as such interventions may confound the effects of snow cover change by introducing additional climatic variables unrelated to snow.

(3) Only farmland experiments based on natural snow regimes were included; studies using artificial snow deposition were excluded.

(4) To minimize environmental confounding, studies conducted in heterogeneous terrains (e.g., slopes or valleys), where snowmelt could be influenced by topography or radiation rather than snow depth, were also excluded.

(5) Studies had to report means, standard deviations, and sample sizes for at least one variable related to plants, soils, or microorganisms.

**Figure 9:**
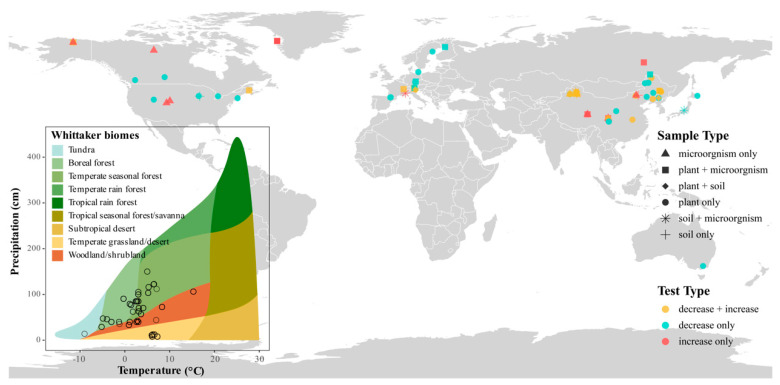
Locations of included studies, with snow manipulation type (color) and sample components (shape). Inset shows biome classification by temperature and precipitation.

A total of 73 peer-reviewed articles published between 2008 and 2024 met the inclusion criteria, yielding 1986 independent observations. Among these, 1047 data points were eligible for pairwise matching, allowing for direct comparisons of coordinated responses across ecosystem components. For each study, we mapped the extracted geographic coordinates (latitude and longitude) alongside mean annual temperature (MAT) and mean annual precipitation (MAP) (Figure 9). In addition, we compiled information on mean annual snowfall, ecosystem type, soil physicochemical properties at different depths, microbial biomass carbon (MBC), microbial biomass nitrogen (MBN), and the type of snow manipulation (e.g., snow addition or removal). Due to data limitations, variables such as the duration of snow manipulation and mean annual snowfall were excluded from further analyses, as they were only reported in approximately 40% of the observations.

To ensure reliability, each article was independently screened by at least two researchers through a two-stage evaluation process. Any discrepancies or ambiguous inclusion decisions were reviewed and resolved by a third researcher. Additionally, we supplemented our dataset by extracting relevant information from existing meta-analyses on snow cover manipulation. For studies that reported only graphical summary data, we retrieved numeric values using the graph calibration tool in Web Plot Digitizer (version 4.2). A full list of studies included in the data extraction process is provided in the Supplementary Information (Literature included in the meta-analysis).

### 4.2. Calculation of the Effect Size

To quantify the effects of snow cover manipulation (i.e., snow addition and snow removal) on plant, soil, and microbial variables—and to evaluate the coupling relationships among them—we calculated the natural logarithm of the response ratio (Ln RR) for each study contrast [42]. The Ln RR is a widely used metric in ecological meta-analyses, representing the proportional change in treatment means on a logarithmic scale [39].

Due to the presence of negative values in some soil temperature data, which preclude logarithmic transformation, we used Hedges’ d—a standardized mean difference suitable for continuous variables measured on different scales—to estimate effect sizes for these cases. The corresponding formulas are as follows:

(1) $$lnRR_{i}=\ln\left(X_{T}\right)-\ln\left(X_{C}\right)$$

(2) $$d=\frac{X_{e}-X_{c}}{\sqrt{\frac{\left(N_{e}-1\right)S_{e}^{2}+(N_{c}-1)S_{c}^{2}}{N_{e}+N_{c}-2}}}J$$

(3) $$J=1-\frac{3}{4(N_{e}+N_{c}-2)}$$

In Equation (1), *X_T_* and *X_C_* are the means under snow manipulation and ambient (control) conditions, respectively. In Equations (2) and (3), *Xe* and *Xc* refer to the means of the experimental and control groups, Se and Sc are their standard deviations, and *Ne* and *Nc* are the respective sample sizes. *J* is a small-sample correction factor.

We also calculated the variance for Ln RR and Hedges’ d using the following equations:

(4) $$V_{i}\left(lnRR_{i}\right)=\frac{S_{T}^{2}}{N_{T}X_{T}^{2}}+\frac{S_{C}^{2}}{N_{C}X_{C}^{2}}$$

(5) $$V_{d}=\frac{N_{e}+N_{c}}{N_{e}N_{c}}+\frac{d^{2}}{2(N_{e}+N_{c})}$$

In Equation (4), *S_T_* and *S_C_* are the standard deviations of the treatment and control groups, and *N_T_* and *N_C_* are their corresponding sample sizes. In Equation (5), *d* is the Hedges’ d effect size calculated above.

When only the standard error (SE) was reported and the standard deviation (SD) was not available, SD was calculated using the following formula:

(6) $$SD=SE\times \sqrt{n}$$

In this meta-analysis, we accounted for both within-study and between-study variance when estimating effect sizes. Each effect size was weighted by the inverse of its total sampling variance [43]. Between-study heterogeneity was estimated using restricted maximum likelihood (REML). To address non-independence among multiple effect sizes from the same study, we treated ‘study’ as a random effect and applied a multi-level mixed-effects meta-analytic model to compute the overall effect sizes while accounting for the hierarchical data structure.

### 4.3. Moderating Variables

In addition to extracting data for effect size calculations, we compiled a suite of moderator variables from selected studies to explore potential sources of heterogeneity in treatment effects. These moderators were chosen based on a priori hypotheses that they may influence the magnitude or direction of ecosystem responses. Specifically, to assess the differential sensitivity of ecosystem components to snow cover change, we examined how plant, soil, and microbial responses varied across ecosystem types and snow manipulation strategies (i.e., snow addition vs. snow removal).

Ecosystem types were categorized into seven classes: grassland, forest, wetland, desert, farmland, tundra, and swamp. We followed the original authors’ qualitative classifications based on methodological descriptions or referenced literature, recognizing that ecosystem characteristics may vary across studies and influence their responses. Snow manipulation approaches were grouped into three categories—shovel snow, snow shed, and snow wall—to test whether treatment method modulates biotic and abiotic responses. To evaluate depth-specific effects on soil and microbial responses, we stratified the soil profile into three layers (0–5 cm, 5–15 cm, and >15 cm), using independently extracted data. Due to wide confidence intervals across all depths for soil temperature, this variable was analyzed separately. Given that snow cover change may affect plants and microbes indirectly through alterations in soil physicochemical properties, we further paired plant aboveground biomass, microbial biomass carbon, and microbial biomass nitrogen with corresponding soil variables within the same studies. These pairwise comparisons enabled us to assess potential linkages between soil properties and above–belowground processes under contrasting snow regimes.

All statistical analyses were conducted in R (version 4.3.3) within RStudio (version 2024.09.0+375, Posit, Boston, MA, USA). Data screening and extraction followed PRISMA guidelines and were carried out using the metagear package [40]. Effect sizes (natural log response ratios, LnRR) and their variances were computed with the metafor package [44], which was also used for random-effects meta-analysis (REML estimation), influence and leave-one-out diagnostics, sensitivity analyses, fail-safe number calculations, funnel plots, Egger’s regression tests, and trim-and-fill procedures to assess and adjust for potential publication bias. To evaluate the relative importance of moderators and explore sources of heterogeneity, we implemented automated model selection and model averaging (AICc criterion) with the glmulti package, ranking predictor variables and visualizing model-averaged importance (Supplementary Figures S5 and S6). Subgroup analyses were further performed for ecosystem type and snow manipulation method—variables consistently reported across all studies—to compare response patterns among ecosystems and manipulation approaches. Post hoc comparisons were conducted using multcomp [45], and all figures (including forest, funnel, and influence plots) were generated with ggplot2 [46] or base R graphics.

### 4.4. Publication Bias

Publication bias refers to the tendency for studies reporting small or non-significant effect sizes to be underrepresented in the published literature, relative to those reporting larger, statistically significant effects [47]. In our analysis, publication bias was assessed where sample sizes allowed, using both Egger’s regression test and funnel plot visualization. For subgroups with a limited number of studies (e.g., *n* < 10), formal assessments of publication bias were not conducted, as these tests have low statistical power under small-sample conditions and are highly sensitive to random variation. In such cases, only forest plots and raw effect size estimates are presented. To evaluate potential publication bias, we examined asymmetry in funnel plots, which display effect sizes against their precision (standard error) (Figures S1 and S2 in the Supporting Information). Egger’s regression test was performed to statistically detect such asymmetry [47]. When the test indicated significant asymmetry (*p* < 0.05), we employed the trim-and-fill method [48] to estimate adjusted effect sizes and assess how conclusions might change if potentially missing studies were imputed (see Figures S3 and S4 in the supporting information). In addition, we conducted sensitivity analyses and corrected for outliers (see Figures S7–S24 in the Supplementary Information). In each figure, panel a shows the sensitivity analysis, while panel b presents the sensitivity analysis after outlier correction to further assess the robustness of the results. In general, results from the trim-and-fill procedure, together with our sensitivity analyses, outlier corrections, and funnel plot assessments, indicated that the overall effect size estimates remained robust. Therefore, we conclude that publication bias is unlikely to significantly affect the interpretation of our findings and that our conclusions are accurate and reliable.

## 5. Conclusions

This study, based on a multi-component meta-analytic framework, deepens our understanding of how snow cover change differentially affects terrestrial ecosystem functioning. Among all measured variables, the effect of snow cover change on soil water content was most pronounced, whereas its impacts on other factors were generally weak. First, it has significant but highly heterogeneous effects across components, with aboveground vegetation and desert systems showing the strongest sensitivity. Second, soil temperature responses were highly variable and strongly context-dependent, shaped by experimental design and background conditions. Finally, correlation analyses among aboveground, belowground, and soil variables revealed asynchronous response patterns, indicating partial decoupling between ecosystem compartments. Building on these findings, this study highlights the need to move beyond isolated component analyses toward more integrated, multi-scale frameworks, and underscores the importance of synchronously examining all ecosystem components under both snow addition and removal scenarios.

## Data Availability

The data presented in this study are available on request from the corresponding author.

## Supplementary Materials

The following supporting information can be downloaded at: https://www.mdpi.com/article/10.3390/plants14203172/s1. Table S1: List of studies included in the meta-analysis, including study ID, biome type, treatment description, and response variables extracted for each ecosystem component. Table S2: Summary statistics of effect sizes (LnRR ± 95% CI) for major ecosystem variables under snow addition and snow removal treatments. Table S3: Moderator variables used in meta-regression analyses and their categorical definitions (e.g., biome, treatment type, duration). Table S4: Results of heterogeneity tests (Q, τ^2^, I^2^) and summary of subgroup analyses for plants, soils, and microbes. Figures S1–S24: Diagnostic plots for publication bias (Egger’s test and funnel plots), sensitivity analyses, and model-averaged importance rankings of moderator variables across ecosystem components under snow addition and snow removal conditions.
