# Supplementary material for: Asynchronous Responses of Plants, Soils, and Microbes to Snow Cover Change Across Terrestrial Ecosystems: A Global Meta-Analysis
Source: Plants (Basel). 2025 Oct 15;14(20):3172. doi: 10.3390/plants14203172 (PMC12566973; doi:10.3390/plants14203172)
Supplement: Supplementary file 1 [file plants-14-03172-s001.zip › plants-3836666-supplementary.pdf]

Appendix:

Literature included in the meta-analysis

1. Zhao, R. *et al.* Winter snowfall can have a positive effect on photosynthetic carbon fixation and biomass accumulation of biological soil crusts from the Gurbantunggut Desert, China. *Ecological research* 31, 251-262 (2016).

2. Gavazov, K. *et al.* Winter ecology of a subalpine grassland: effects of snow removal on soil respiration, microbial structure and function. *Science of the Total Environment* 590, 316-324 (2017).

3. Hui, R. *et al*. Variation in snow cover drives differences in soil properties and microbial biomass of BSCs in the Gurbantunggut Desert - 3 years of snow manipulations. *Ecohydrology* 12, e2118 (2019).

4. Yin, J.-F. *et al*. Typical ephemeral plant—Erodium oxyrhinchum: growth response to snow change in temperate desert, Northwest China. *Journal of Plant Ecology* 16, rtac079 (2023).

5. Lu, Y. *et al.* Stimulating effects of snow cover on gaseous nitrogen emissions are intensified by biological soil crusts. *Geoderma* 432, 116425 (2023).

6. Hosokawa, N. *et al.* Soil freeze–thaw with root litter alters N transformations during the dormant season in soils under two temperate forests in northern Japan. *Soil Biology and Biochemistry* 114, 270-278 (2017).

7. Ricketts, M. P. *et al*. Soil bacterial community and functional shifts in response to altered snowpack in moist acidic tundra of northern Alaska. *Soil* 2, 459-474 (2016).

8. Boswell, E. P. *et al.* Soil aggregation returns to a set point despite seasonal response to snow manipulation. *Geoderma* 357, 113954 (2020).

9. Yang, Y. *et al*. Snow-cover loss attenuates the effects of N addition on desert nutrient cycling and microbial community. *Frontiers in Plant Science* 14, 1166897 (2023).

10. Tan, B. *et al*. Snow removal alters soil microbial biomass and enzyme activity in a Tibetan alpine forest. *Applied Soil Ecology* 76, 34-41 (2014).

11. Zhang, L. *et al.* Snow exclusion does not affect soil ammonia-oxidizing bacteria and archaea communities. *Forests* 13, 1483 (2022).

12. Wang, X. *et al.* Snow depths' impact on soil microbial activities and carbon dioxide fluxes

from a temperate wetland in Northeast China. *Scientific Reports* 10, 8709 (2020).

13.Robroek, B. J. *et al.* Snow cover manipulation effects on microbial community structure and soil chemistry in a mountain bog. *Plant and soil* 369, 151-164 (2013).

14. Fan, L. *et al.* Snow and rainfall independently affect the density, composition and productivity of ephemerals in a temperate desert. *Science of the Total Environment* 807, 151033 (2022).

15. Gao, D. *et al.* Small and transient response of winter soil respiration and microbial communities to altered snow depth in a mid-temperate forest. *Applied Soil Ecology* 130, 40-49 (2018).

16. Yang, F. *et al.* Short-term winter snow reduction stimulates soil nutrient leaching without changing the microbial biomass in an alpine fir forest. *Global Ecology and Conservation* 25, e01434 (2021).

17. Xu, H. *et al*. Short-term snow removal alters fungal but not bacterial beta diversity and structure during the spring snowmelt period in a meadow steppe of China. *Journal of Fungi* 8, 234 (2022).

18. Ren, Y. *et al.* Short-term effects of snow cover manipulation on soil bacterial diversity and community composition. *Science of the Total Environment* 741, 140454 (2020).

19. Yin, S. *et al.* Seasonal response of soil microbial community structure and life history strategies to winter snow cover change in a temperate forest. *Science of The Total Environment* 949, 175066 (2024).

20. Wu, Q. Season-dependent effect of snow depth on soil microbial biomass and enzyme activity in a temperate forest in Northeast China. *Catena* 195, 104760 (2020).

21. Ji, X. *et al.* Response of soil microbial diversity and functionality to snow removal in a cool-temperate forest. *Soil Biology and Biochemistry* 196, 109515 (2024).

22. Zhang, A. *et al.* Plants alter their aboveground and belowground biomass allocation and affect community-level resistance in response to snow cover change in Central Asia, Northwest China. *Science of The Total Environment* 902, 166059 (2023).

23. Baptist, F. *et al.* No increase in alpine snowbed productivity in response to experimental lengthening of the growing season. *Plant Biology* 12, 755-764 (2010).

24. Power, C. C. *et al.* No effect of snow on shrub xylem traits: Insights from a snow-

manipulation experiment on Disko Island, Greenland. *Science of the Total Environment* 916, 169896 (2024).

25. Wang, Z. *et al.* Mutual feeding mechanism of carbon, nitrogen and enzyme activity in Northeast China black soil under snow cover change. *Applied Soil Ecology* 190, 104991 (2023).
26. Bombonato, L. *et al.* Manipulating snow cover in an alpine bog: effects on ecosystem respiration and nutrient content in soil and microbes. *Climatic Change* 114, 261-272 (2012).
27. Morgado, L. N. *et al.* Long-term increase in snow depth leads to compositional changes in arctic ectomycorrhizal fungal communities. *Global Change Biology* 22, 3080-3096 (2016).
28. Christiansen, C. T. *et al.* Long-term deepened snow promotes tundra evergreen shrub growth and summertime ecosystem net CO 2 gain but reduces soil carbon and nutrient pools. *Global Change Biology* 24, 3508-3525 (2018).
29. Fan, L. L. *et al.* The limited role of snow water in the growth and development of ephemeral plants in a cold desert. *Journal of Vegetation Science* 25, 681-690 (2014).
30. Ma, W. *et al.* Later-melting rather than thickening of snowpack enhance the productivity and alter the community composition of temperate grassland. *Science of The Total Environment* 923, 171440 (2024).
31. Henry, H. A. *et al.* Increased soil frost versus summer drought as drivers of plant biomass responses to reduced precipitation: results from a globally coordinated field experiment. *Ecosystems* 21, 1432-1444 (2018).
32. Stark, S. *et al.* Ice-on-snow and compacted and absent snowpack exert contrasting effects on soil carbon cycling in a northern boreal forest. *Soil Biology and Biochemistry* 150, 107983 (2020).
33. Voříšková, J. *et al.* Fast response of fungal and prokaryotic communities to climate change manipulation in two contrasting tundra soils. *Environmental Microbiome* 14, 6 (2019).
34. Chimner, R. *et al.* Experimental manipulations of winter snow and summer rain influence ecosystem carbon cycling in a mixed-grass prairie, Wyoming, USA. *Ecohydrology* 3, 284-293 (2010).
35. Zhao, R. *et al.* Effects of snowfall depth on soil physical–chemical properties and soil

microbial biomass in moss–dominated crusts in the Gurbantunggut Desert, Northern China. *Catena* 169, 175-182 (2018).

36. Liu, L. *et al*. Effects of snow removal on seasonal dynamics of soil bacterial community and enzyme activity. *European Journal of Soil Biology* 119, 103564 (2023).
37. Li, Z. *et al.* Effects of snow absence on winter soil nitrogen dynamics in a subalpine spruce forest of southwestern China. *Geoderma* 307, 107-113 (2017).
38. Gaul, D. *et al*. Effects of experimental soil frost on the fine-root system of mature Norway spruce. *Journal of Plant Nutrition and Soil Science* 171, 690-698 (2008).
39. Ade, L. *et al.* Effect of snowpack on the soil bacteria of alpine meadows in the Qinghai-Tibetan Plateau of China. *Catena* 164, 13-22 (2018).
40. Hui, R. *et al*. Effect of snow cover on water content, carbon and nutrient availability, and microbial biomass in complexes of biological soil crusts and subcrust soil in the desert. *Geoderma* 406, 115505 (2022).
41. Mao, W. *et al.* Divergent interactive impacts on productivity and functional diversity from fluctuated snowfall and continuous nitrogen pollution within Inner Mongolian. *Science of the Total Environment* 704, 135443 (2020).
42. Jia, Z. *et al.* Deepened snow loosens temporal coupling between plant and microbial N utilization and induces ecosystem N losses. *Global Change Biology* 28, 4655-4667 (2022).
43. Xu, W. *et al.* Deepened snow enhances gross nitrogen cycling among Pan-Arctic tundra soils during both winter and summer. *Soil Biology and Biochemistry* 160, 108356 (2021).
44. Deng, M. *et al.* Deepened snow cover increases grassland soil carbon stocks by incorporating carbon inputs into deep soil layers. *Global Change Biology* 29, 4686-4696 (2023).
45. Sorensen, P. O. *et al*. Contrasting effects of winter snowpack and soil frost on growing season microbial biomass and enzyme activity in two mixed-hardwood forests. *Biogeochemistry* 128, 141-154 (2016).
46. Gasarch, E. I. *et al*. The consequences of multiple resource shifts on the productivity and composition of alpine tundra communities: inferences from a long-term snow and nutrient manipulation experiment. *Plant Ecology & Diversity* 8, 751-761 (2015).
47. Suzuki, R. O. Combined effects of warming, snowmelt timing, and soil disturbance on

vegetative development in a grassland community. *Plant Ecology* 215, 1399-1408 (2014).

48. Fan, L. *et al*. Combined effects of snow depth and nitrogen addition on ephemeral growth at the southern edge of the Gurbantunggut Desert, China. *Journal of Arid Land* 5, 500-510 (2013).

49. Broadbent, A. A. D. *et al.* Climate change alters temporal dynamics of alpine soil microbial functioning and biogeochemical cycling via earlier snowmelt. *The ISME Journal*, 15(8): 2264-2275 (2021).

50. Hermesdorf, L. *et al.* Changes in soil and plant carbon pools after 9 years of experimental summer warming and increased snow depth. *Science of The Total Environment* 951, 175648 (2024).

51. Brin, L. D. *et al*. Changes in snow cover alter nitrogen cycling and gaseous emissions in agricultural soils. *Agriculture, Ecosystems & Environment* 258, 91-103 (2018).

52. Kreyling, J. *et al*. Absence of snow cover reduces understory plant cover and alters plant community composition in boreal forests. *Oecologia* 168, 577-587 (2012).

53. Blume-Werry, G. *et al*. Short-term climate change manipulation effects do not scale up to long-term legacies: effects of an absent snow cover on boreal forest plants. *Journal of Ecology,* 104, 1638-1648 (2016).

54. Christiansen, C. T. *et al.* Long-term deepened snow promotes tundra evergreen shrub growth and summertime ecosystem net CO_2 gain but reduces soil carbon and nutrient pools. *Global Change Biology,* 24, 3508-3525 (2018).

55. D'Imperio, L. *et al.* Fast Responses of Root Dynamics to Increased Snow Deposition and Summer Air Temperature in an Arctic Wetland. *Frontiers in Plant Science,* 9 (2018).

56. Domisch, T. *et al.* Let it snow! Winter conditions affect growth of birch seedlings during the following growing season. *Tree Physiology,* 39, 544-555 (2019).

57. Luo, X. et al. Response of soil microbial function diversity to snow cover gradient in alpine meadow soil of QinghaiTibet Plateau. *Journal of Glaciology and Geocryology*, 40 (5) : 1016 -1027 (2018). (In Chinese with English Abstract)

58. Tang, C. Response of alpine meadows in permafrost regions of the Qinghai－Tibet Plateau to increased snow cover. *Master's thesis*, Sichuan Agricultural University, Sichuan, China (2021). (In Chinese with English Abstract)

59. Liu, YQ. Effects of snow cover change on soil respiration and microbial communities during the nongrowing season in temperate grasslands. *Journal of Natural Geography*, (2022). (In Chinese with English Abstract)

60. Yan, J. W. Effects of snow cover change on soil organic carbon sequestration and its mechanisms in peat bogs of Northeast China. *Master ' s thesis*, Northeast Normal University, Changchun, China (2024). (In Chinese with English Abstract)

61. Li, Z. J. et al. Effects of snow removal on soil active nitrogen in subalpine spruce forests in western Sichuan. *Chinese Journal of Applied Ecology*, 28, 1786 (2017). (In Chinese with English Abstract)

62. Ma, D. L. *et al*. Effects of snow cover changes on soil microbial community structure in permafrost regions of the Greater Khingan Mountains. *Acta Ecologica Sinica*, 40, 789 – 799 (2020). (In Chinese with English Abstract)

63. Tan, X. J. *et al*. Effects of increased snow cover on soil phosphorus availability in alpine meadows of the Qinghai – Tibet Plateau. *Acta Prataculturae Sinica*, 33 (2024). (In Chinese with English Abstract)

64. Wang, E. L. *et al*. Seasonal effects of snow cover changes on soil soluble carbon and nitrogen content and microbial activity. *Chinese Journal of Eco-Agriculture*, 31, 1976 – 1983 (2023). (In Chinese with English Abstract)

65. Wang, Z. L. *et al.* Effects of snow cover changes on base cations and bioavailable silicon and aluminum in black soil of northeastern farmlands. *Journal of Soil and Water Conservation*, 38, 147 – 156 (2024). (In Chinese with English Abstract)

66. Wang, K. Feedback mechanisms between available carbon and nitrogen and enzyme activities under changing snow cover in black soils. *Doctoral dissertation*, Northeast Agricultural University, Harbin, China (2023). (In Chinese with English Abstract)

67. Zan, Z. M. *et al.* Effects of extreme snowfall events on soil respiration in plantation forests of the semi-arid to warm-temperate transitional zone of North Asia. *Journal of Forestry Research*, 33, 27 – 34 (2020). (In Chinese with English Abstract)

68. Wang, X. Responses of plant community and root to snowpack change in an alpine meadow of Northwestern Sichuan, China. *Master's dissertation*, Southwest Minzu University, Chengdu, China (2020). (In Chinese with English Abstract)

69. CUI, F X. *et al*. Responses of soil bacterial composition and diversity to snow cover in cold temperate larch forests. *Journal of Central South University of Forestry & Technology*, 43(1):132-143 (2023). (In Chinese with English Abstract)

70. Fan, L L. *et al*. Response of the herbaceous layer to snow variability at the south margin of the Gurbantong-gut Desert of China. *Chinese Journal of Plant Ecology,* 36 (2): 126–135 (2012). (In Chinese with English Abstract)

71. Mao, J. *et al*. Influences of warming and snow reduction in winter on soil nutrients and bacterial communi-ties composition in a typical grassland of the Loess Plateau. *Chinese Journal of Plant Ecology*, 45 (8): 891-902 (2021). (In Chinese with English Abstract)

72. Mao, J. The Influence of Warming or Snow Reduction During the Dormancy Period on Soil Physicochemical Properties and Bacterial Communities in Loess Plateau. *Master's dissertation*, Northwest A&F University, College of Grassland Agriculture China (2021). (In Chinese with English Abstract)

73. Huang, XJ. *et al*. Soil Biomass Carbon Dynamics of Subalpine Forest in Western Sichuan Province during the Cold Seaso. *JOURNAL OF MOUNTAIN SCIENCE*, 30, 543-549 (2012). (In Chinese with English Abstract)

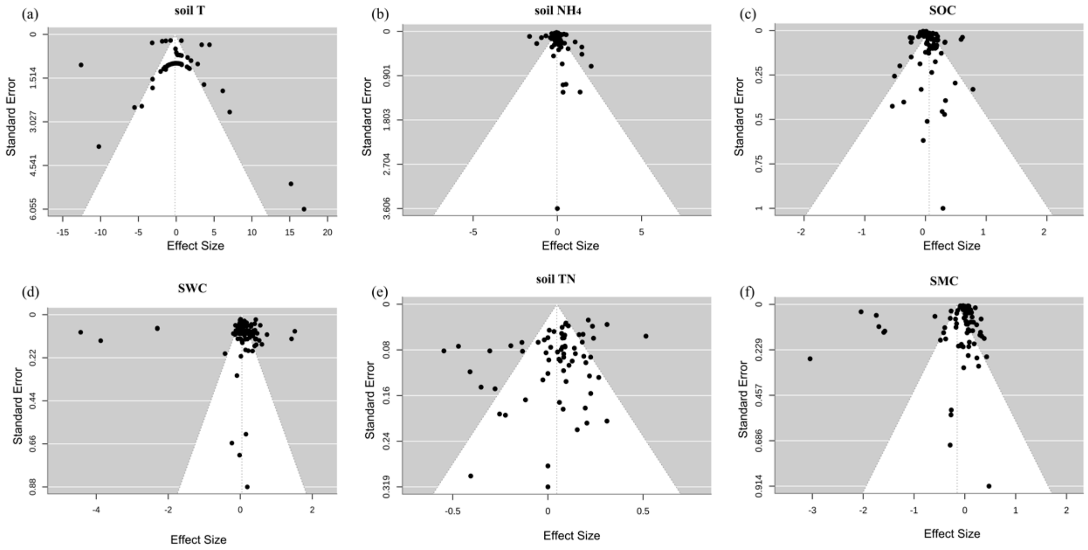

Figure S1. Funnel plots indicating no evidence of publication bias under snow addition treatments. Funnel plots display the relationship between effect size and standard error for soil temperature (soil T), ammonium (soil NH_4), soil organic carbon (SOC), soil water content (SWC), soil total nitrogen (TN), and soil microbial biomass carbon/nitrogen (SMC/SMN) under snow addition treatments. Egger's regression tests showed no significant funnel plot asymmetry for any of the variables: soil T ($z = 1.0701$, $p = 0.2846$), soil NH_4 ($z = 1.3000$, $p = 0.1936$), SOC ($z = 0.4394$, $p = 0.6604$), SWC ($z = -0.0655$, $p = 0.9478$), soil TN ($z = -1.7378$, $p = 0.0822$), SMC ($z = -0.2839$, $p = 0.7765$). These results suggest no publication bias in the snow addition subset for these indicators.

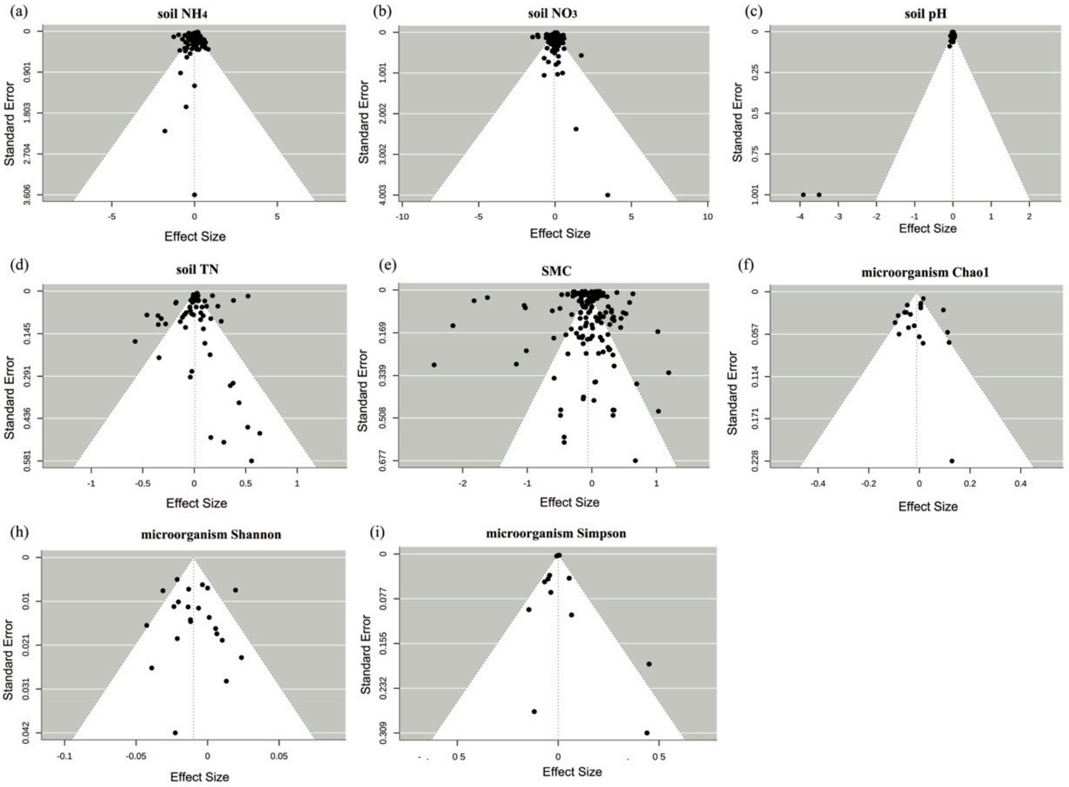

Figure S2. Funnel plots indicating no evidence of publication bias under snow reduction treatments. Funnel plots display the effect size versus standard error under snow removal treatments for the following variables: soil NH_4, soil nitrate (soil NO_3), soil pH, soil TN, SMC, microbial richness index (Chao1), microbial Shannon index, and microbial Simpson index. Egger's regression tests indicated no significant funnel plot asymmetry: soil NH_4 ($z = 0.0372$, $p = 0.9703$), soil NO_3 ($z = 1.5676$, $p = 0.1170$), soil pH ($z = -1.6707$, $p = 0.0948$), soil TN ($z = 0.2548$, $p = 0.7989$), SMC ($z = 0.6904$, $p = 0.4899$), microorganism Chao1 ($z = 0.5169$, $p = 0.6053$), Shannon ($z = 0.3004$, $p = 0.7639$), and Simpson ($z = -0.1665$, $p = 0.8678$).These results suggest no publication bias for these indicators under snow removal conditions.

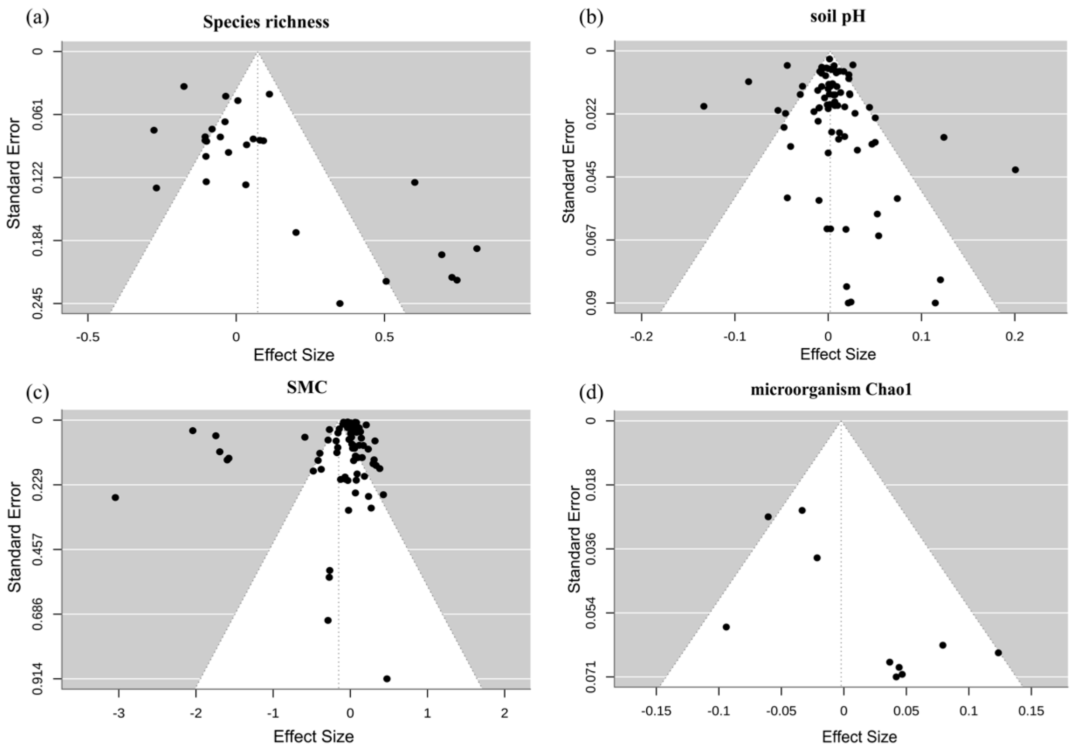

Figure S3. Funnel plots under snow addition treatments showing significant publication bias for selected variables. Funnel plots illustrate the relationship between effect size and standard error for species richness, soil pH, microbial biomass nitrogen (SMC), and microbial Chao1 richness under snow addition conditions. Egger's regression tests revealed significant funnel plot asymmetry for: species richness ($z = 5.399$, $p < 0.0001$), soil ammonium ($z = 2.6915$, $p = 0.0071$), soil pH ($z = 2.0833$, $p = 0.0372$), microbial biomass nitrogen ($z = 3.2653$, $p = 0.0011$), microbial Chao1 richness ($z = 2.7836$, $p = 0.0054$). The asymmetry observed in several panels suggests potential publication bias, which was further evaluated and corrected using the trim-and-fill method. Despite these indications of asymmetry, results from the trim-and-fill procedure indicated that the overall effect size estimates remained robust. Therefore, we conclude that publication bias is unlikely to significantly affect the interpretation of our findings.

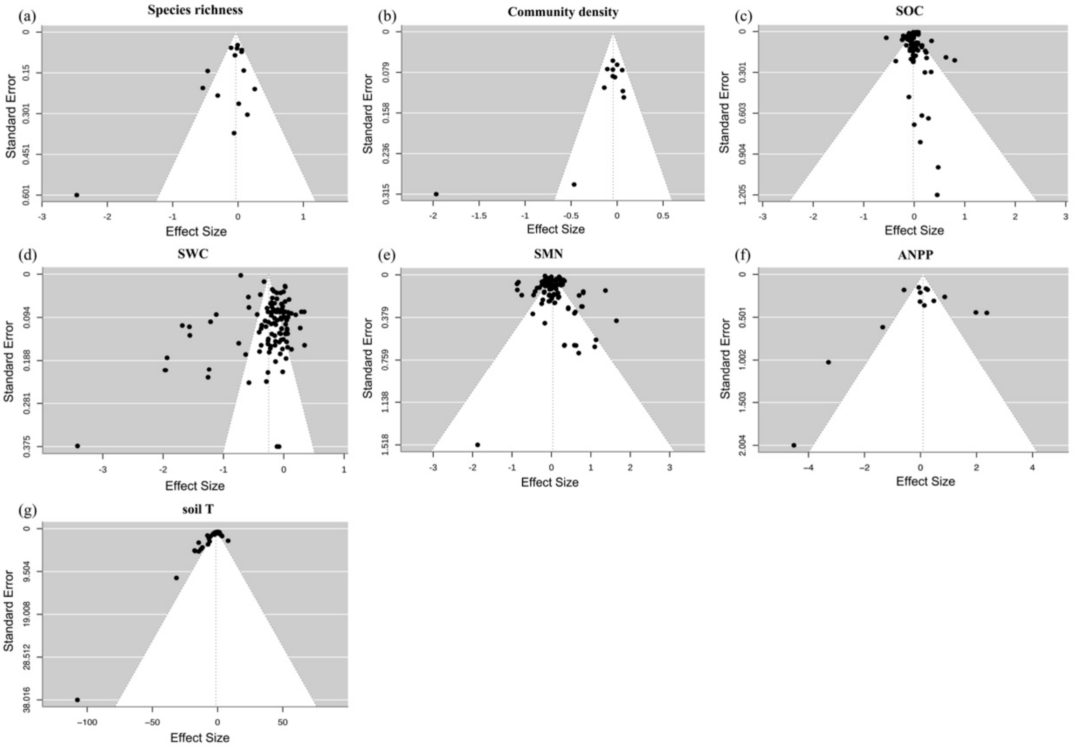

Figure S4. Funnel plots under snow removal treatments showing significant publication bias for multiple ecosystem indicators. Funnel plots show the relationship between effect size and standard error for species richness, community density, aboveground net primary productivity (ANPP), SOC, SWC, SMN, and soil T under snow removal conditions. Egger's regression tests detected significant funnel plot asymmetry for: species richness ($z = -2.7069$, $p = 0.0068$), community density ($z = -4.3586$, $p < 0.0001$), ANPP ($z = -2.5372$, $p = 0.0112$), SOC ($z = 2.0411$, $p = 0.0412$), SWC ($z = -4.1678$, $p < 0.0001$), SMN ($z = 2.8559$, $p = 0.0043$), soil T ($z = -8.7584$, $p < 0.0001$). The asymmetry observed in several panels suggests potential publication bias, which was further evaluated and corrected using the trim-and-fill method. Despite these indications of asymmetry, results from the trim-and-fill procedure indicated that the overall effect size estimates remained robust. Therefore, we conclude that publication bias is unlikely to significantly affect the interpretation of our findings.

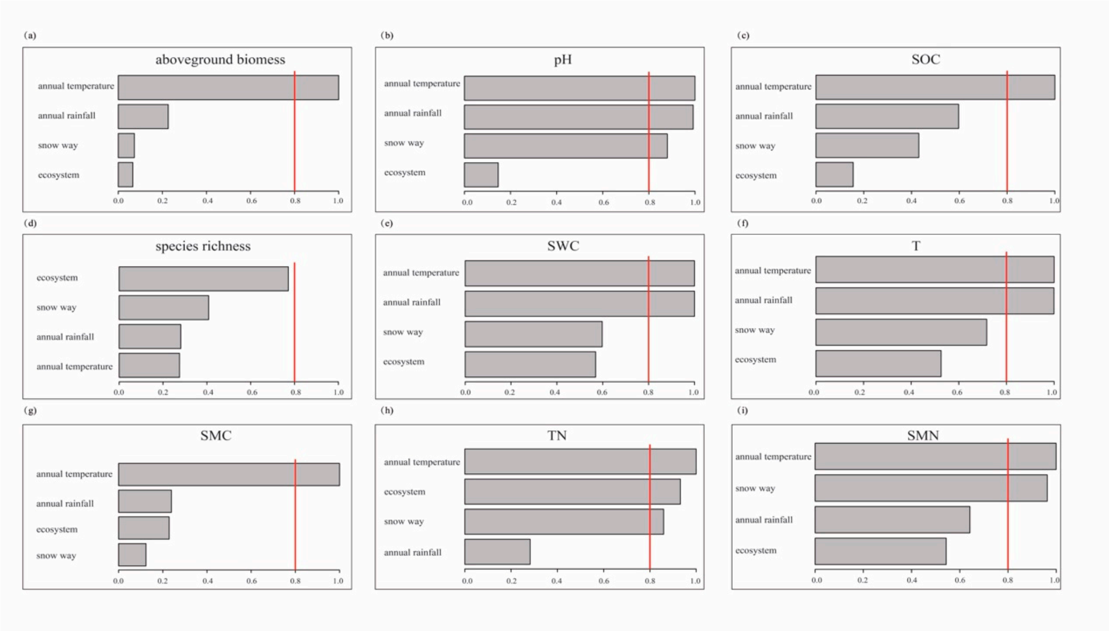

Figure S5. Model-averaged importance of predictor variables for multiple ecosystem indicators under snow addition treatments. Bars show the relative contribution of annual temperature, annual rainfall, snow manipulation method, and ecosystem type to explaining variation in (a) aboveground biomass, (b) pH, (c) SOC, (d) species richness, (e) SWC, (f) soil temperature (T), (g) SMC, (h) TN, and (i) SMN. The vertical red line in each panel indicates the threshold for high relative importance (≥0.8). Although climatic factors (annual temperature and annual rainfall) generally exert stronger influence than snow manipulation method or ecosystem type under snow addition conditions, these two variables were not included as explanatory variables in the main analysis because data coverage was limited to about 40%.

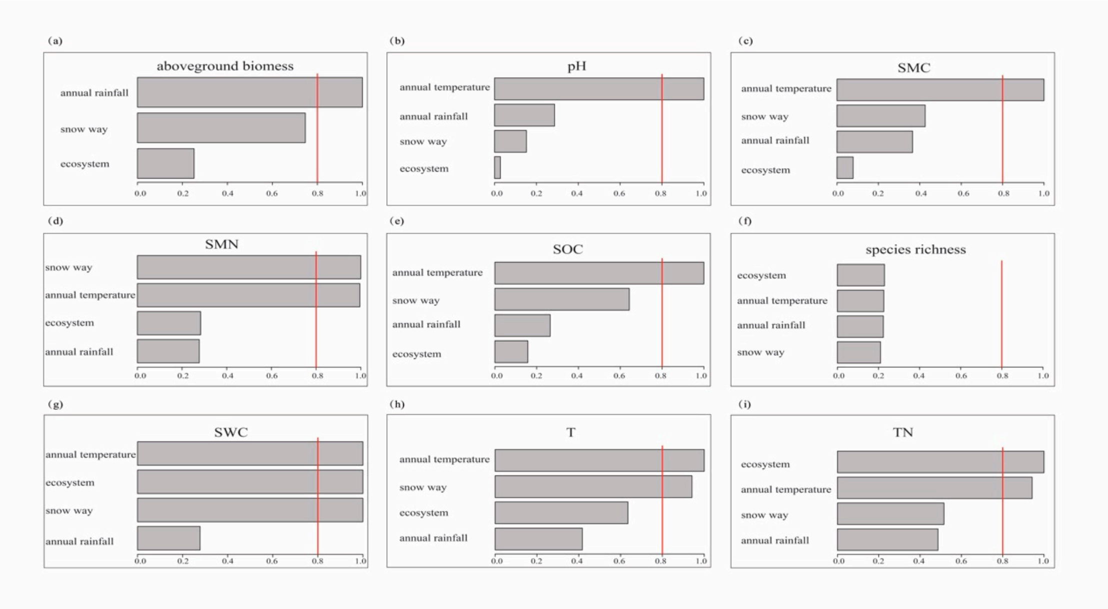

Figure S6. Model-averaged importance of predictor variables for multiple ecosystem indicators under snow removal treatments. Bars show the relative contribution of annual temperature, annual rainfall, snow manipulation method, and ecosystem type to explaining variation in (a) aboveground biomass, (b) pH, (c) SMC, (d) SMN, (e) SOC, (f) species richness, (g) SWC, (h) soil temperature (T), and (i) TN. The vertical red line in each panel indicates the threshold for high relative importance (≥0.8). Although climatic factors (annual temperature and annual rainfall) generally exert stronger influence than snow manipulation method or ecosystem type under snow removal conditions, these two variables were not included as explanatory variables in the main analysis because data coverage was limited to about 40%.

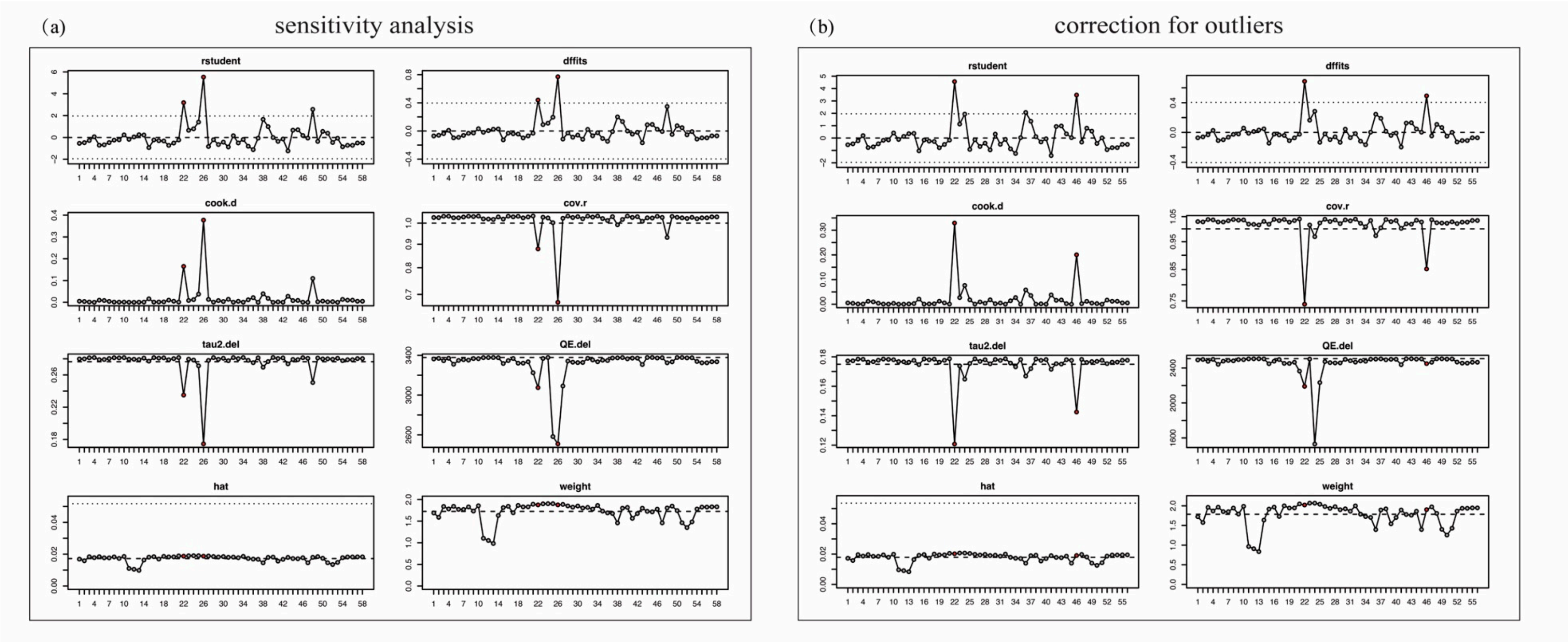

Figure S7. Sensitivity analysis for the meta-analysis of aboveground biomass responses to snow addition. Panel (a) shows influence diagnostics from a random-effects model: studentized residuals (rstudent), DFFITS, Cook's distance (cook.d), covariance ratio (cov.r), leave-one-out change in between-study variance (tau2.del), leave-one-out change in residual heterogeneity (QE.del), leverage (hat), and study weights (weight). Dashed lines mark reference thresholds; red points denote observations flagged as potentially influential/outlying. Panel (b) displays the same diagnostics after correcting for outliers by removing the flagged observations from panel (a). The stabilization of cov.r, tau2.del, QE.del, and weight profiles indicates that the overall effect estimate is robust to these studies, supporting the consistency of our main conclusions.

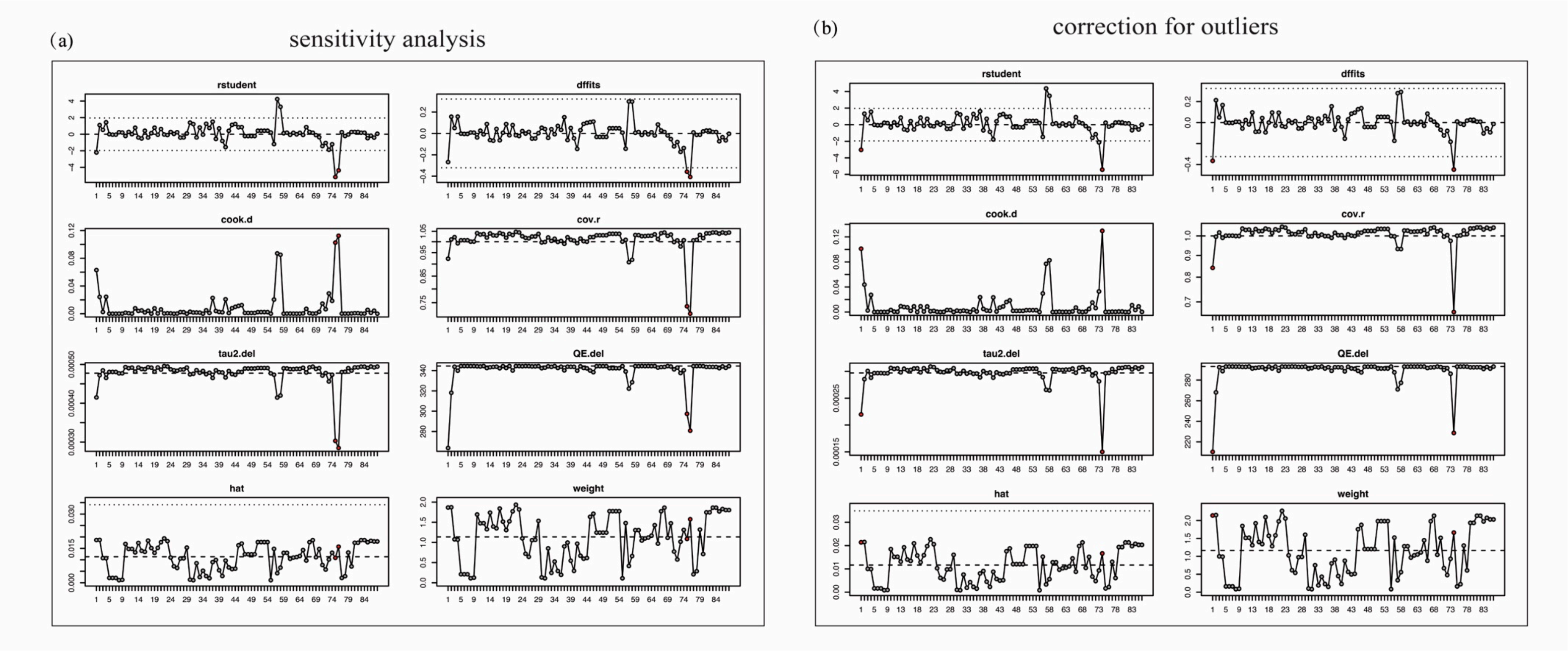

Figure S8. Sensitivity analysis for the meta-analysis of soil pH responses to snow addition. Panel (a) shows influence diagnostics from a random-effects model: studentized residuals (rstudent), DFFITS, Cook's distance (cook.d), covariance ratio (cov.r), leave-one-out change in between-study variance (tau2.del), leave-one-out change in residual heterogeneity (QE.del), leverage (hat), and study weights (weight). Dashed lines mark reference thresholds; red points denote observations flagged as potentially influential/outlying. Panel (b) displays the same diagnostics after correcting for outliers by removing the flagged observations from panel (a). The stabilization of cov.r, tau2.del, QE.del, and weight profiles indicates that the overall effect estimate for pH is robust to these studies, supporting the consistency of our main conclusions.

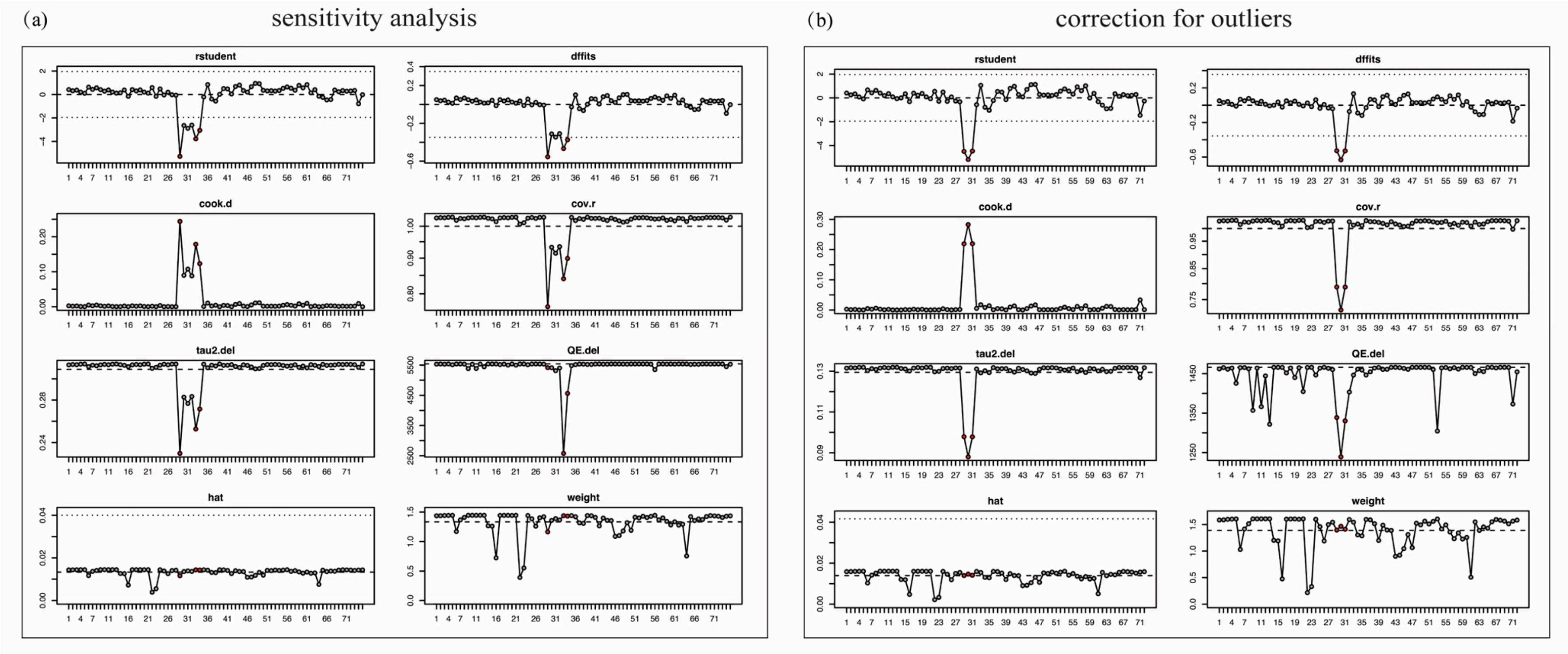

Figure S9. Sensitivity analysis for the meta-analysis of soil microbial biomass carbon (SMC) responses to snow addition. Panel (a) shows influence diagnostics from a random-effects model: studentized residuals (rstudent), DFFITS, Cook's distance (cook.d), covariance ratio (cov.r), leave-one-out change in between-study variance (tau2.del), leave-one-out change in residual heterogeneity (QE.del), leverage (hat), and study weights (weight). Dashed lines mark reference thresholds; red points denote observations flagged as potentially influential/outlying. Panel (b) displays the same diagnostics after correcting for outliers by removing the flagged observations from panel (a). The stabilization of cov.r, tau2.del, QE.del, and weight profiles indicates that the overall effect estimate for SMC is robust to these studies, supporting the consistency of our main conclusions.

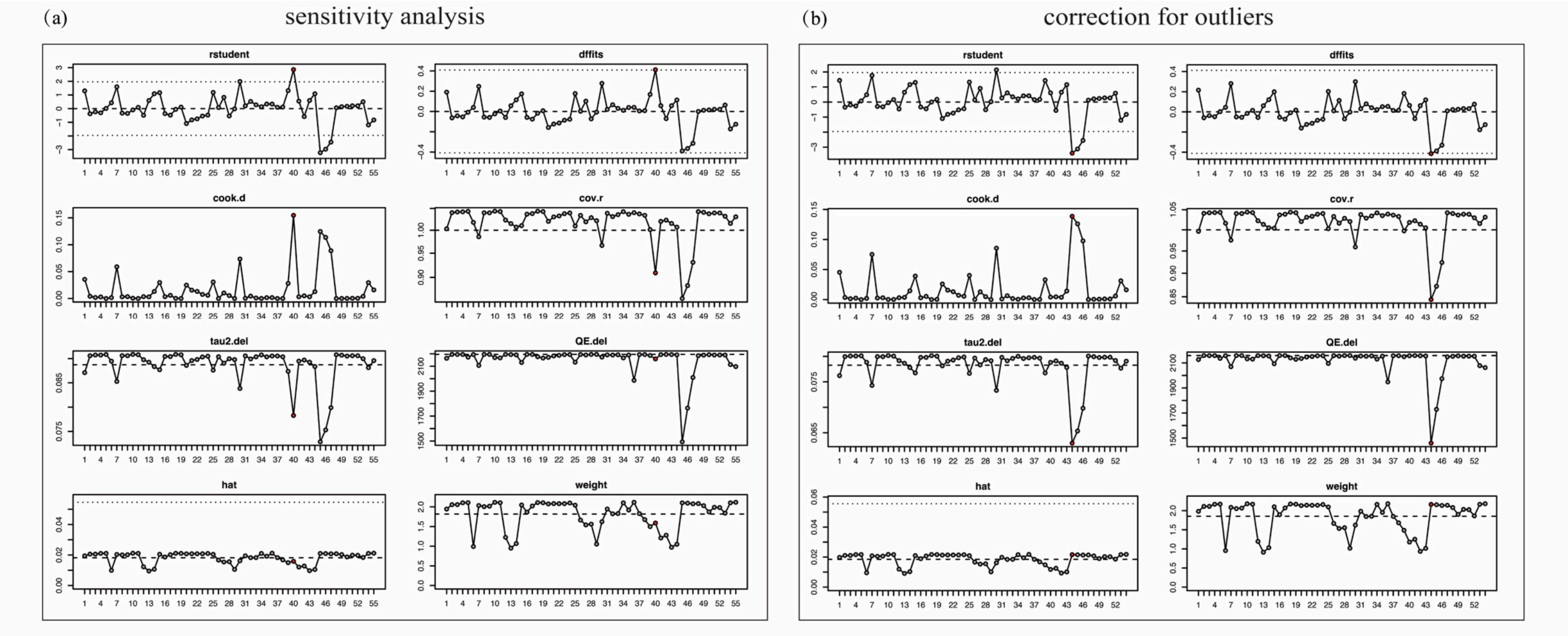

Figure S10. Sensitivity analysis for the meta-analysis of soil microbial biomass nitrogen (SMN) responses to snow addition. Panel (a) shows influence diagnostics from a random-effects model: studentized residuals (rstudent), DFFITS, Cook's distance (cook.d), covariance ratio (cov.r), leave-one-out change in between-study variance (tau2.del), leave-one-out change in residual heterogeneity (QE.del), leverage (hat), and study weights (weight). Dashed lines mark reference thresholds; red points denote observations flagged as potentially influential/outlying. Panel (b) displays the same diagnostics after correcting for outliers by removing the flagged observations from panel (a). The stabilization of cov.r, tau2.del, QE.del, and weight profiles indicates that the overall effect estimate for SMN is robust to these studies, supporting the consistency of our main conclusions.

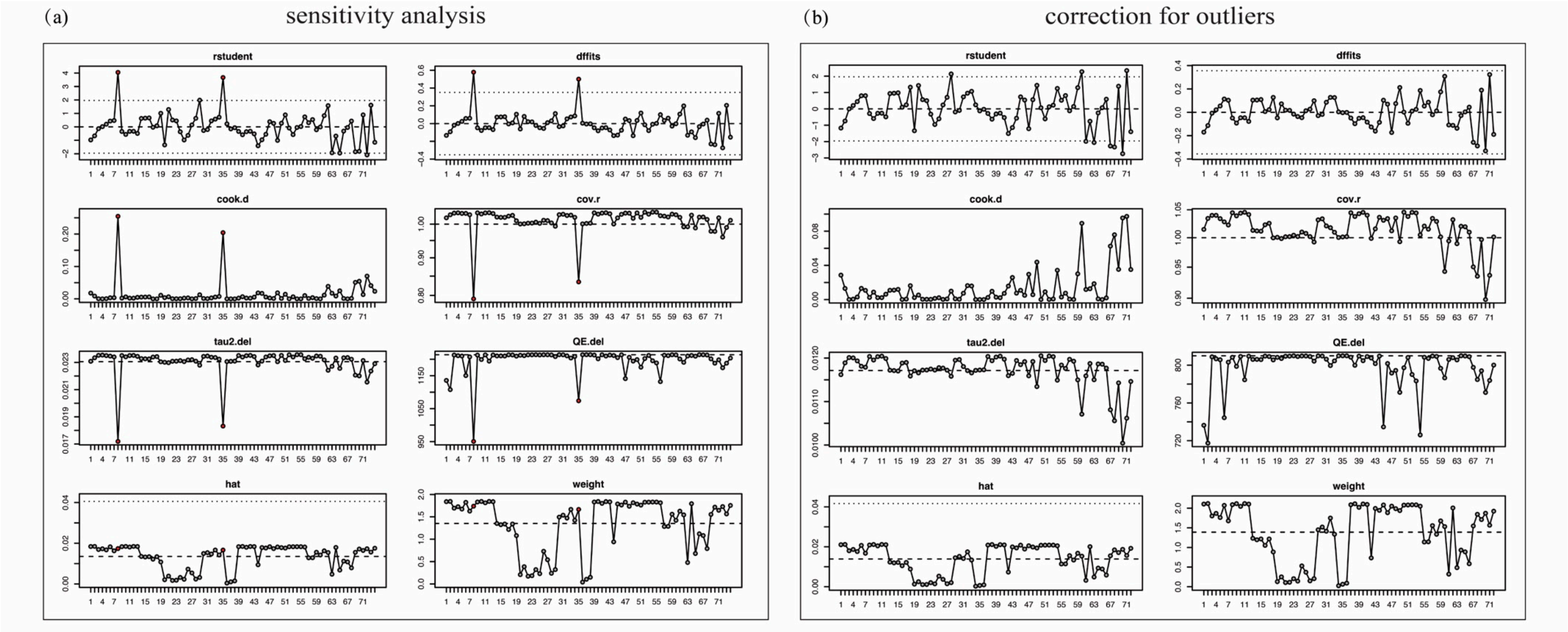

Figure S11. Sensitivity analysis for the meta-analysis of soil organic carbon (SOC) responses to snow addition. Panel (a) shows influence diagnostics from a random-effects model: studentized residuals (rstudent), DFFITS, Cook's distance (cook.d), covariance ratio (cov.r), leave-one-out change in between-study variance (tau2.del), leave-one-out change in residual heterogeneity (QE.del), leverage (hat), and study weights (weight). Dashed lines mark reference thresholds; red points denote observations flagged as potentially influential/outlying. Panel (b) displays the same diagnostics after correcting for outliers by removing the flagged observations from panel (a). The stabilization of cov.r, tau2.del, QE.del, and weight profiles indicates that the overall effect estimate for SOC is robust to these studies, supporting the consistency of our main conclusions.

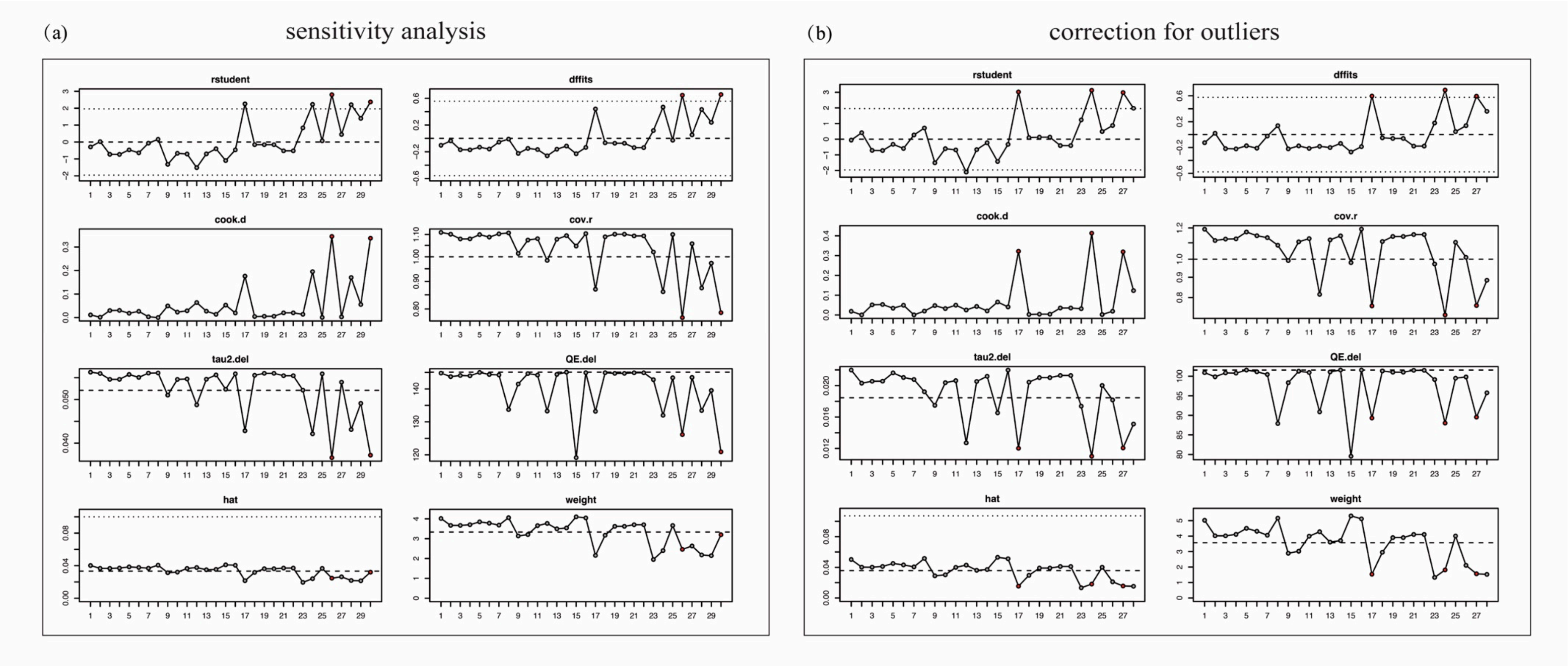

Figure S12. Sensitivity analysis for the meta-analysis of species richness responses to snow addition. Panel (a) shows influence diagnostics from a random-effects model: studentized residuals (rstudent), DFFITS, Cook's distance (cook.d), covariance ratio (cov.r), leave-one-out change in between-study variance (tau2.del), leave-one-out change in residual heterogeneity (QE.del), leverage (hat), and study weights (weight). Dashed lines mark reference thresholds; red points denote observations flagged as potentially influential/outlying. Panel (b) displays the same diagnostics after correcting for outliers by removing the flagged observations from panel (a). The stabilization of cov.r, tau2.del, QE.del, and weight profiles indicates that the overall effect estimate for species richness is robust to these studies, supporting the consistency of our main conclusions.

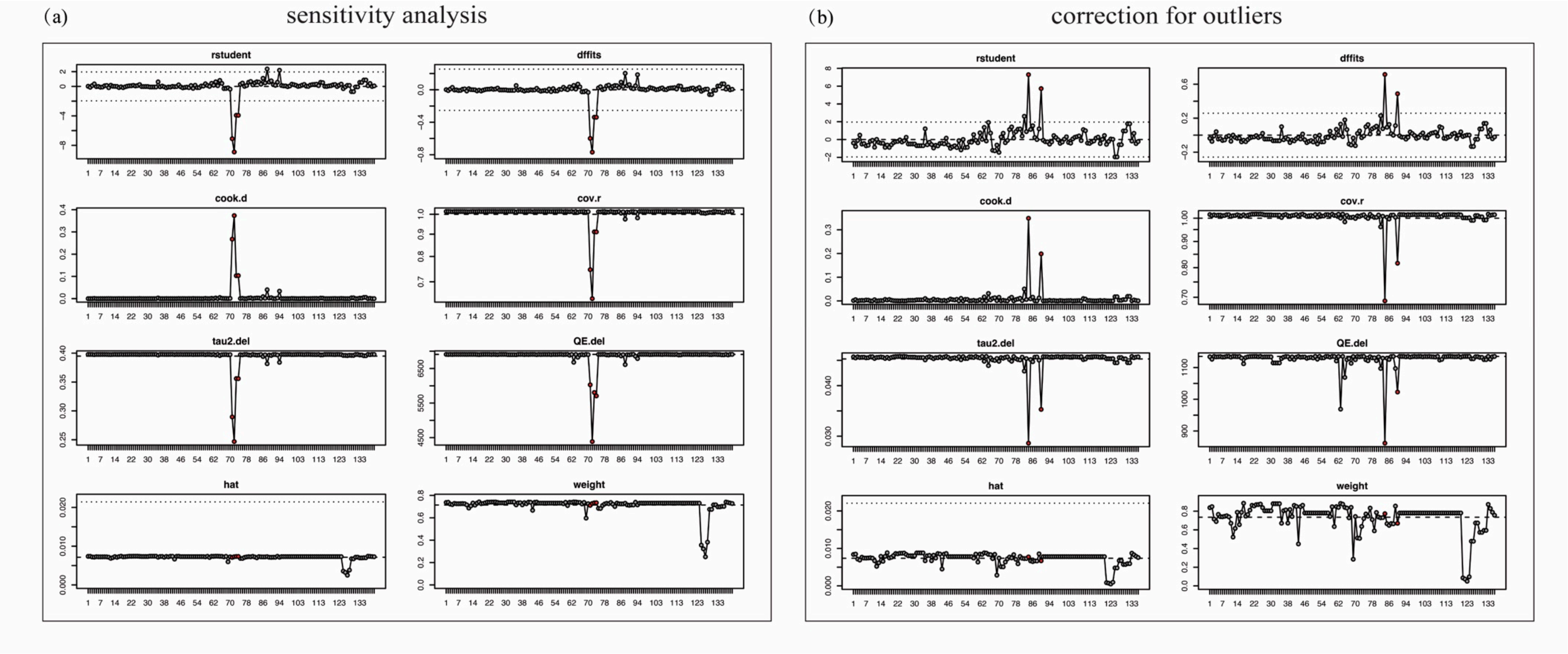

Figure S13. Sensitivity analysis for the meta-analysis of soil water content (SWC) responses to snow addition. Panel (a) shows influence diagnostics from a random-effects model: studentized residuals (rstudent), DFFITS, Cook's distance (cook.d), covariance ratio (cov.r), leave-one-out change in between-study variance (tau2.del), leave-one-out change in residual heterogeneity (QE.del), leverage (hat), and study weights (weight). Dashed lines mark reference thresholds; red points denote observations flagged as potentially influential/outlying. Panel (b) displays the same diagnostics after correcting for outliers by removing the flagged observations from panel (a). The stabilization of cov.r, tau2.del, QE.del, and weight profiles indicates that the overall effect estimate for SWC is robust to these studies, supporting the consistency of our main conclusions.

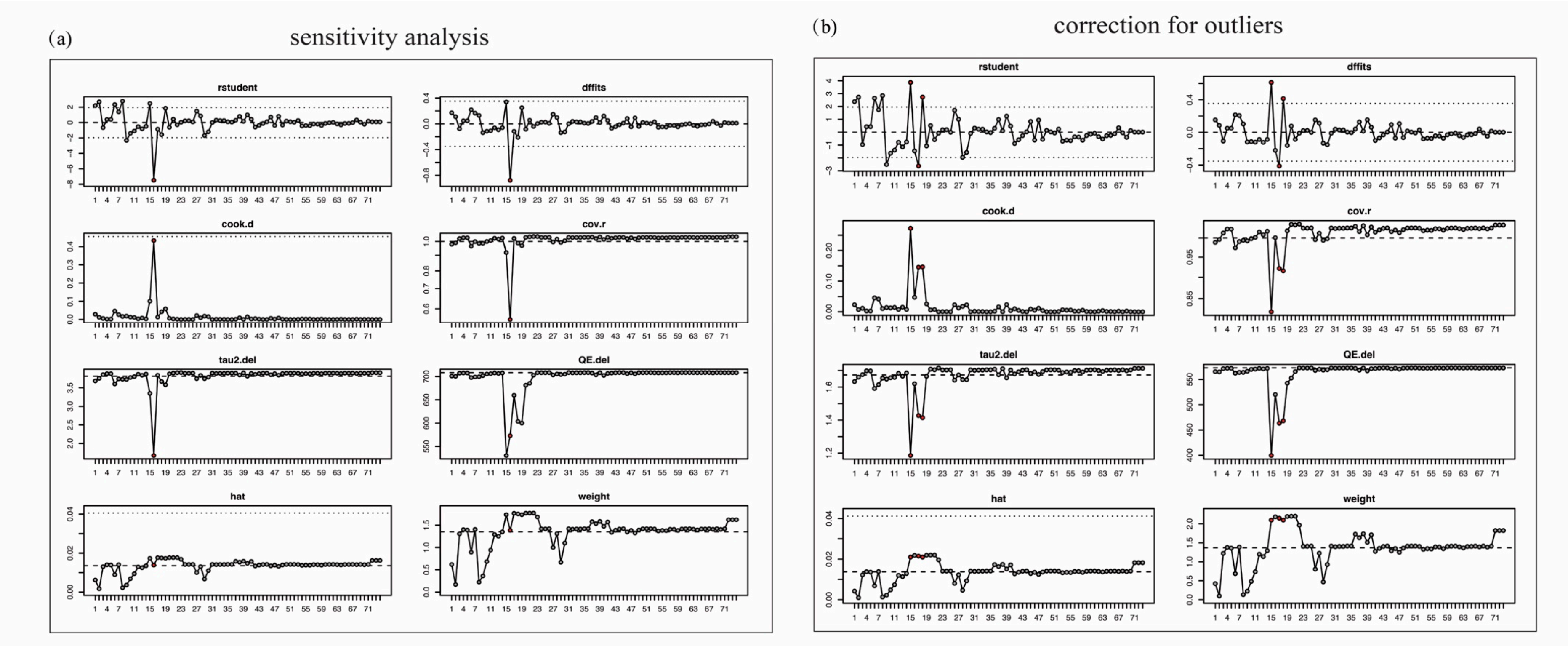

Figure S14. Sensitivity analysis for the meta-analysis of soil temperature (T) responses to snow addition. Panel (a) shows influence diagnostics from a random-effects model: studentized residuals (rstudent), DFFITS, Cook's distance (cook.d), covariance ratio (cov.r), leave-one-out change in between-study variance (tau2.del), leave-one-out change in residual heterogeneity (QE.del), leverage (hat), and study weights (weight). Dashed lines mark reference thresholds; red points denote observations flagged as potentially influential/outlying. Panel (b) displays the same diagnostics after correcting for outliers by removing the flagged observations from panel (a). The stabilization of cov.r, tau2.del, QE.del, and weight profiles indicates that the overall effect estimate for T is robust to these studies, supporting the consistency of our main conclusions.

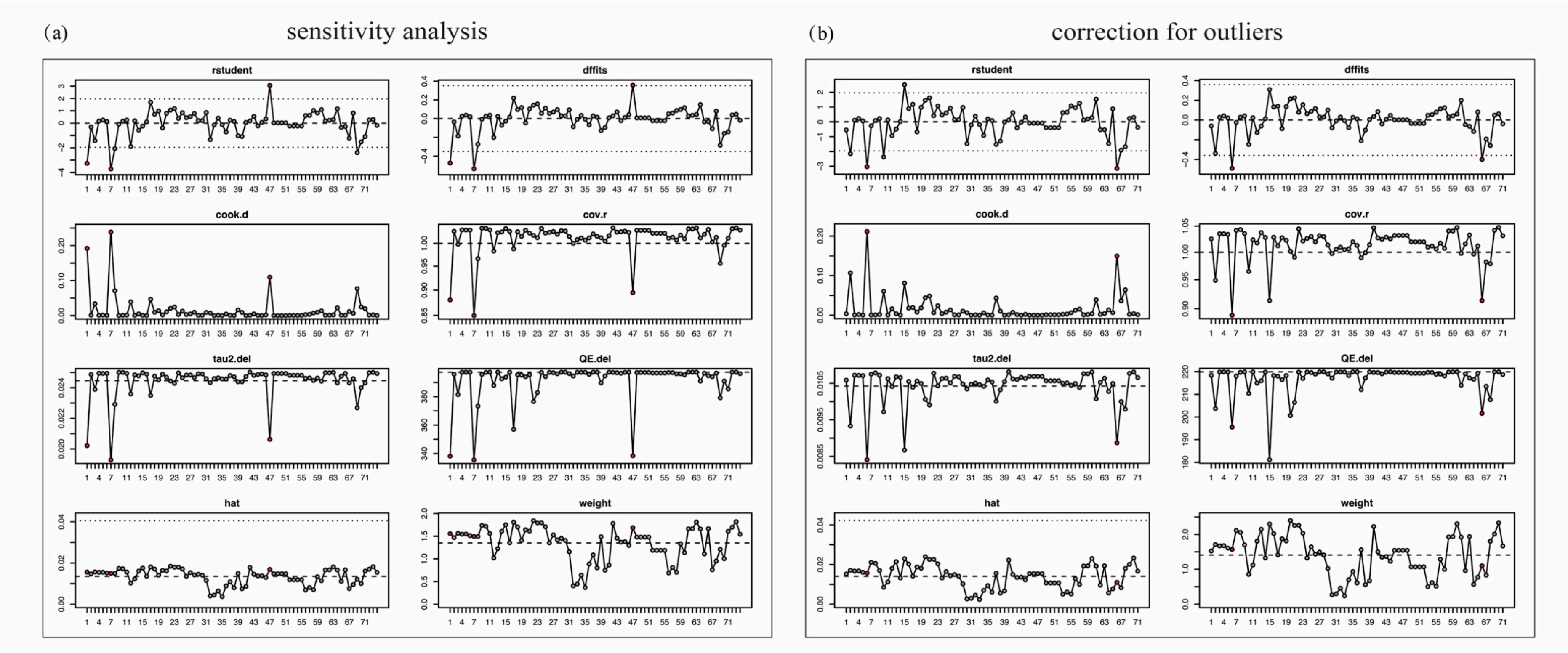

Figure S15. Sensitivity analysis for the meta-analysis of soil total nitrogen (TN) responses to snow addition. Panel (a) shows influence diagnostics from a random-effects model: studentized residuals (rstudent), DFFITS, Cook's distance (cook.d), covariance ratio (cov.r), leave-one-out change in between-study variance (tau2.del), leave-one-out change in residual heterogeneity (QE.del), leverage (hat), and study weights (weight). Dashed lines mark reference thresholds; red points denote observations flagged as potentially influential/outlying. Panel (b) displays the same diagnostics after correcting for outliers by removing the flagged observations from panel (a). The stabilization of cov.r, tau2.del, QE.del, and weight profiles indicates that the overall effect estimate for TN is robust to these studies, supporting the consistency of our main conclusions.

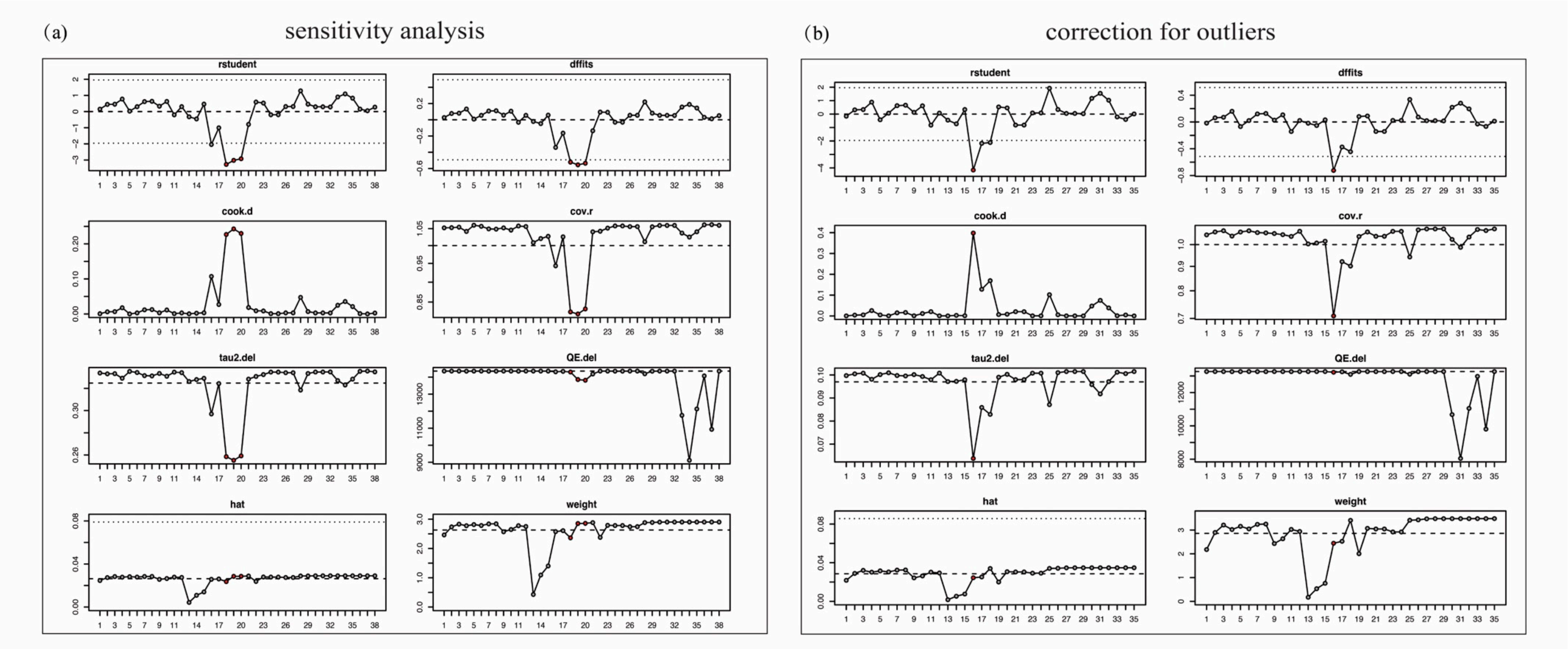

Figure S16. Sensitivity analysis for the meta-analysis of aboveground biomass responses to snow removal. Panel (a) shows influence diagnostics from a random-effects model: studentized residuals (rstudent), DFFITS, Cook's distance (cook.d), covariance ratio (cov.r), leave-one-out change in between-study variance (tau2.del), leave-one-out change in residual heterogeneity (QE.del), leverage (hat), and study weights (weight). Dashed lines mark reference thresholds; red points denote observations flagged as potentially influential/outlying. Panel (b) displays the same diagnostics after correcting for outliers by removing the flagged observations from panel (a). The stabilization of cov.r, tau2.del, QE.del, and weight profiles indicates that the overall effect estimate for aboveground biomass is robust to these studies, supporting the consistency of our main conclusions.

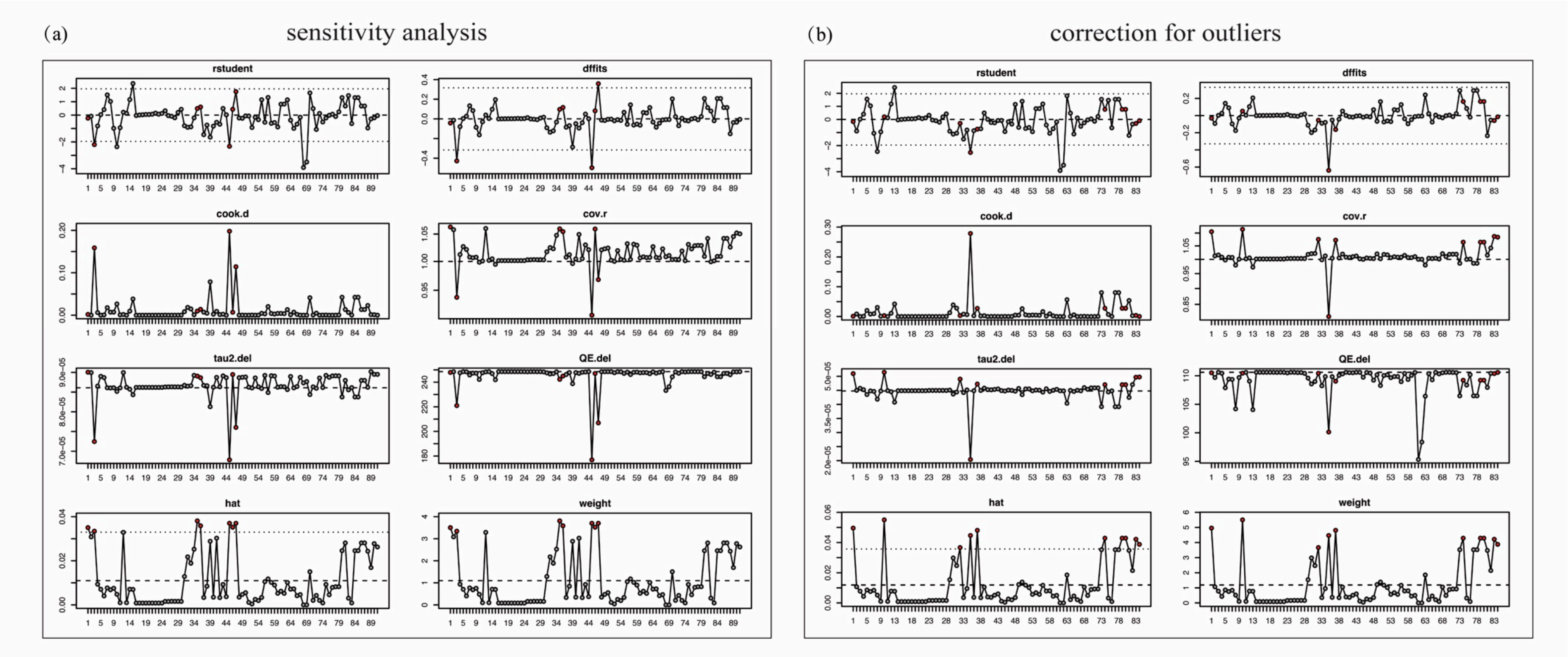

Figure S17. Sensitivity analysis for the meta-analysis of soil pH responses to snow removal. Panel (a) shows influence diagnostics from a random-effects model: studentized residuals (rstudent), DFFITS, Cook's distance (cook.d), covariance ratio (cov.r), leave-one-out change in between-study variance (tau2.del), leave-one-out change in residual heterogeneity (QE.del), leverage (hat), and study weights (weight). Dashed lines mark reference thresholds; red points denote observations flagged as potentially influential/outlying. Panel (b) displays the same diagnostics after correcting for outliers by removing the flagged observations from panel (a). The stabilization of cov.r, tau2.del, QE.del, and weight profiles indicates that the overall effect estimate for pH is robust to these studies, supporting the consistency of our main conclusions.

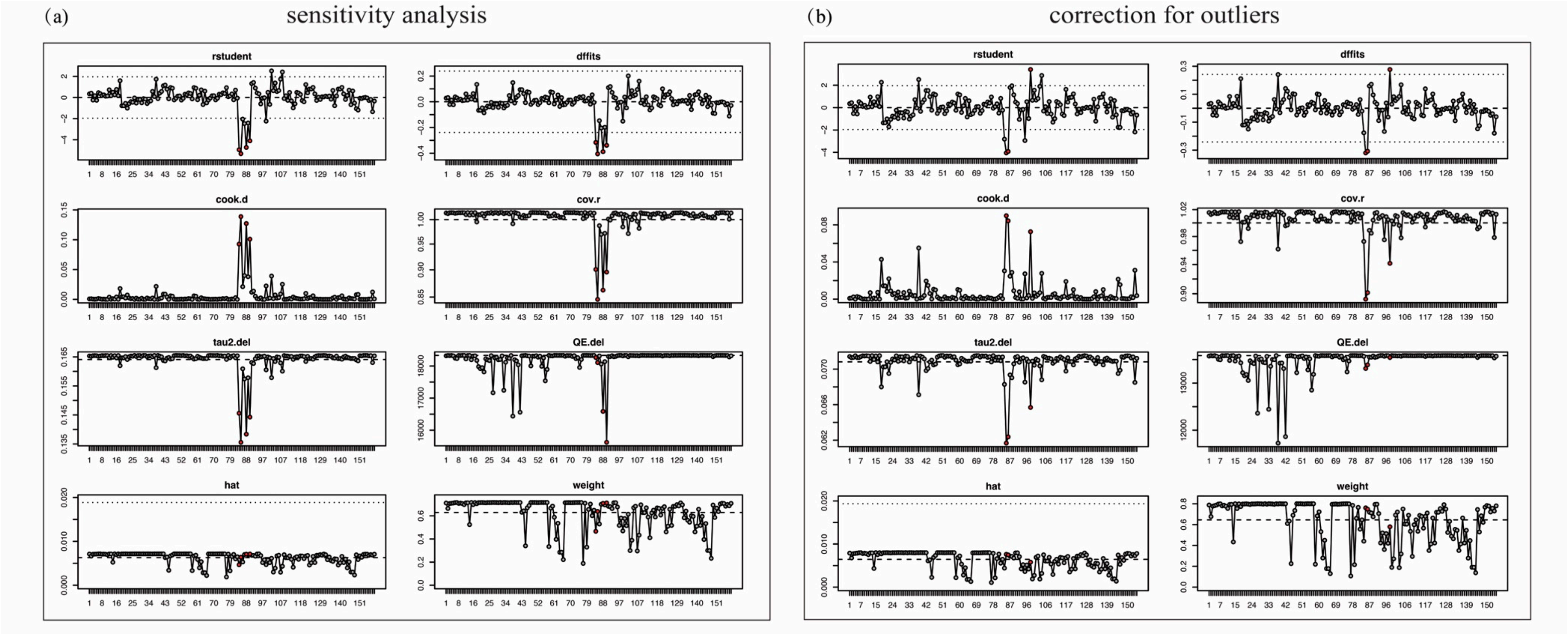

Figure S18. Sensitivity analysis for the meta-analysis of soil microbial biomass carbon (SMC) responses to snow removal. Panel (a) shows influence diagnostics from a random-effects model: studentized residuals (rstudent), DFFITS, Cook's distance (cook.d), covariance ratio (cov.r), leave-one-out change in between-study variance (tau2.del), leave-one-out change in residual heterogeneity (QE.del), leverage (hat), and study weights (weight). Dashed lines mark reference thresholds; red points denote observations flagged as potentially influential/outlying. Panel (b) displays the same diagnostics after correcting for outliers by removing the flagged observations from panel (a). The stabilization of cov.r, tau2.del, QE.del, and weight profiles indicates that the overall effect estimate for SMC is robust to these studies, supporting the consistency of our main conclusions.

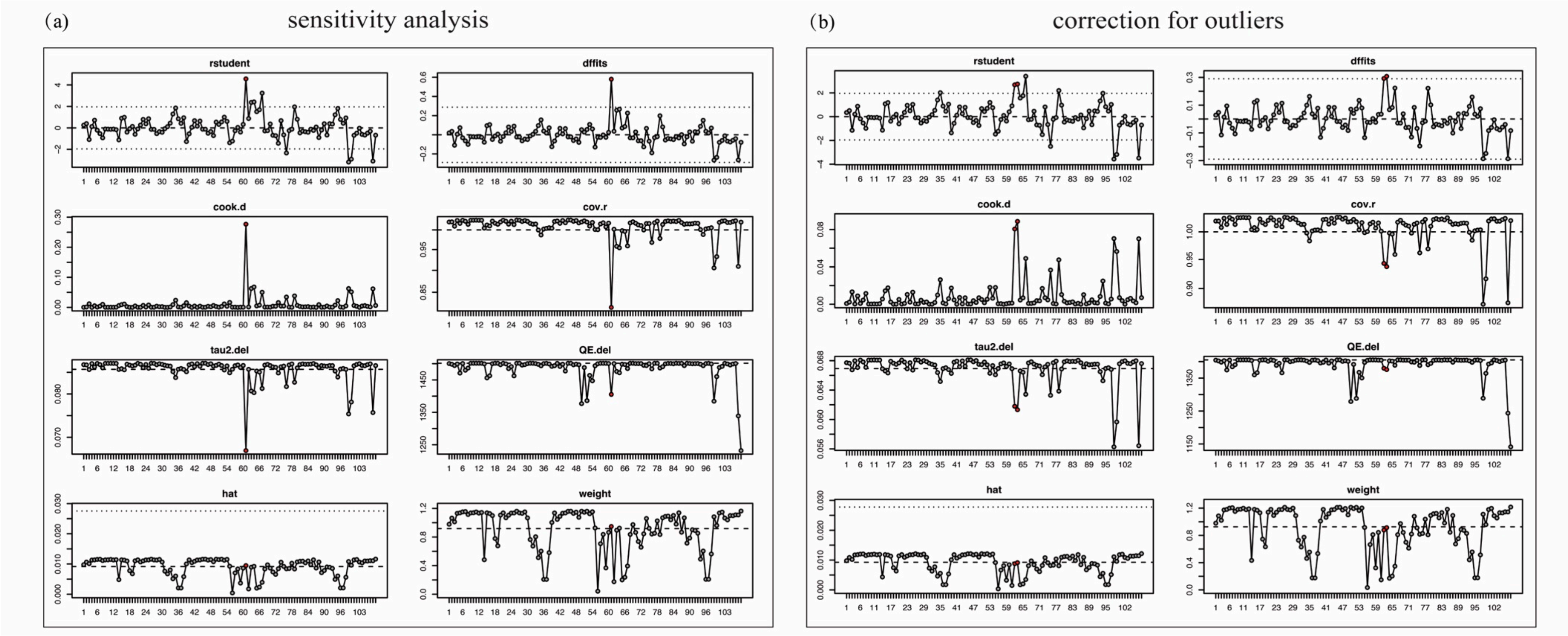

Figure S19. Sensitivity analysis for the meta-analysis of soil microbial biomass nitrogen (SMN) responses to snow removal. Panel (a) shows influence diagnostics from a random-effects model: studentized residuals (rstudent), DFFITS, Cook's distance (cook.d), covariance ratio (cov.r), leave-one-out change in between-study variance (tau2.del), leave-one-out change in residual heterogeneity (QE.del), leverage (hat), and study weights (weight). Dashed lines mark reference thresholds; red points denote observations flagged as potentially influential/outlying. Panel (b) displays the same diagnostics after correcting for outliers by removing the flagged observations from panel (a). The stabilization of cov.r, tau2.del, QE.del, and weight profiles indicates that the overall effect estimate for SMN is robust to these studies, supporting the consistency of our main conclusions.

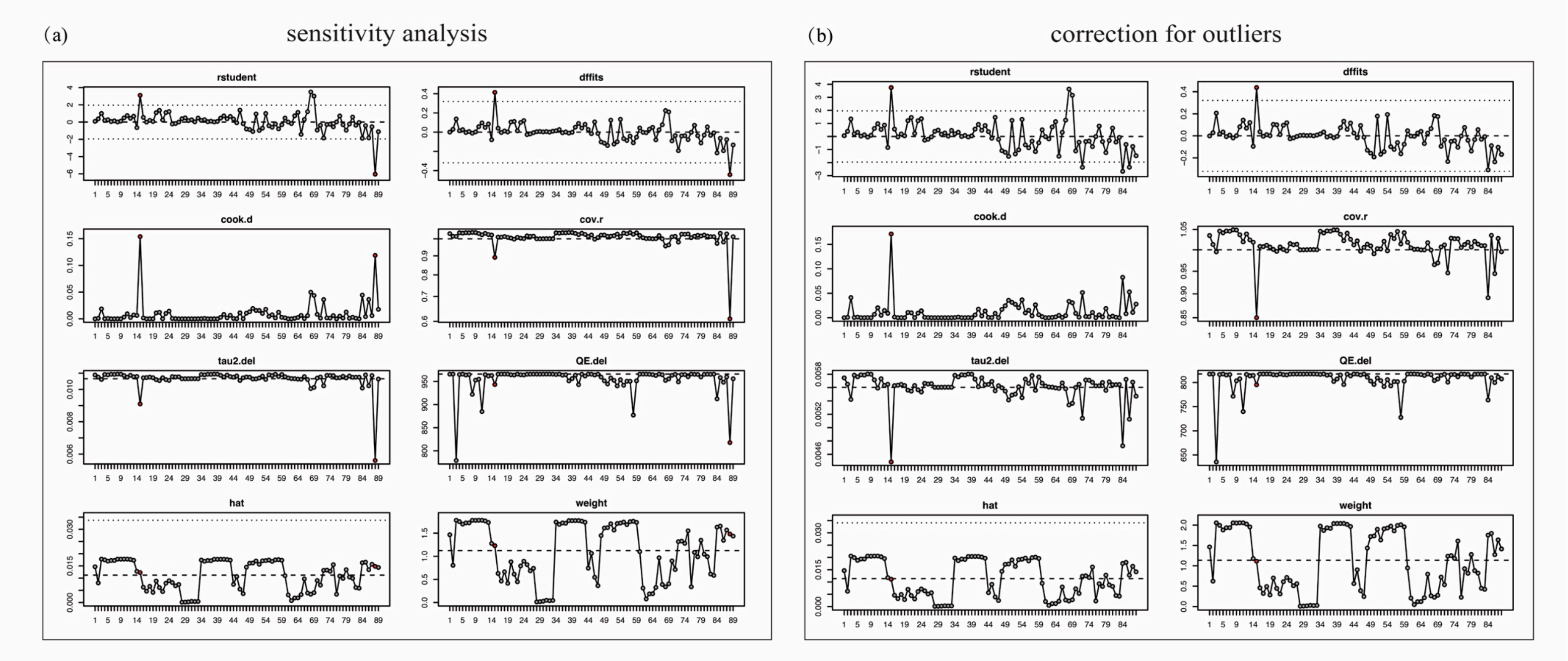

Figure S20. Sensitivity analysis for the meta-analysis of soil organic carbon (SOC) responses to snow removal. Panel (a) shows influence diagnostics from a random-effects model: studentized residuals (rstudent), DFFITS, Cook's distance (cook.d), covariance ratio (cov.r), leave-one-out change in between-study variance (tau2.del), leave-one-out change in residual heterogeneity (QE.del), leverage (hat), and study weights (weight). Dashed lines mark reference thresholds; red points denote observations flagged as potentially influential/outlying. Panel (b) displays the same diagnostics after correcting for outliers by removing the flagged observations from panel (a). The stabilization of cov.r, tau2.del, QE.del, and weight profiles indicates that the overall effect estimate for SOC is robust to these studies, supporting the consistency of our main conclusions.

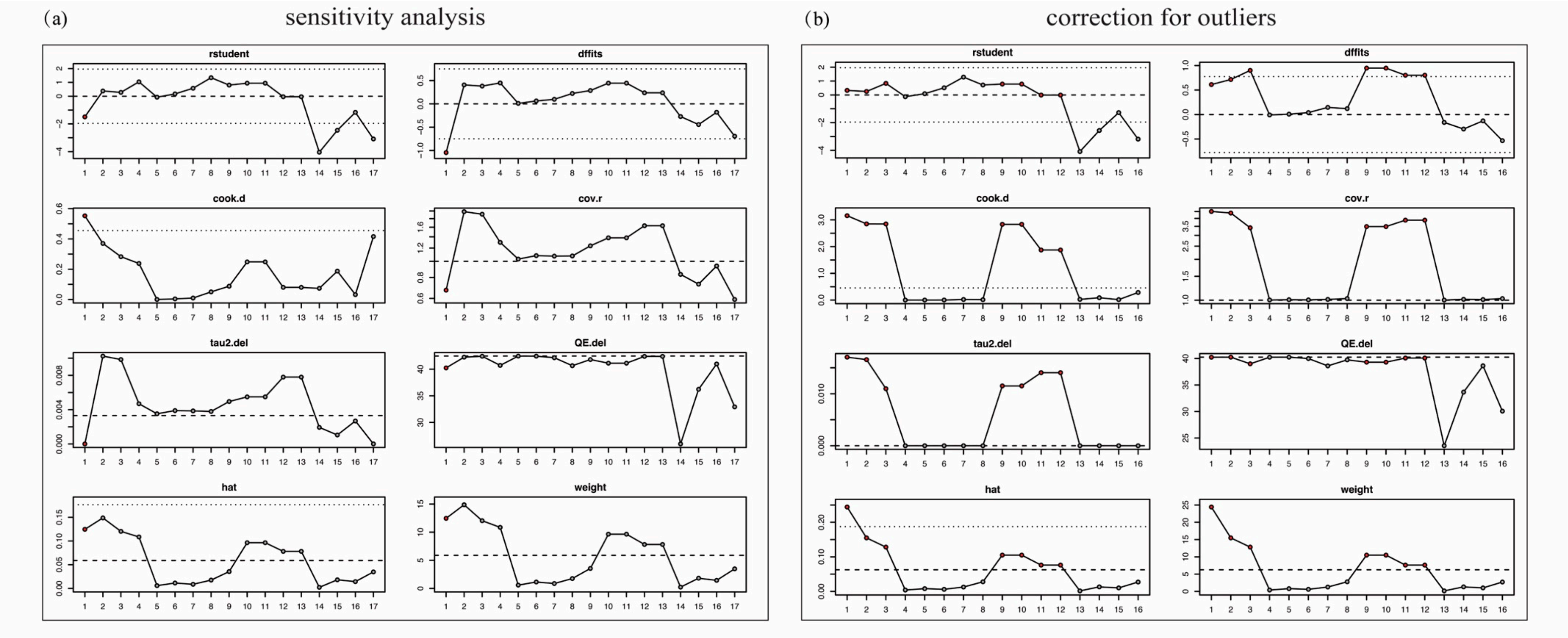

Figure S21. Sensitivity analysis for the meta-analysis of species richness responses to snow removal. Panel (a) shows influence diagnostics from a random-effects model: studentized residuals (rstudent), DFFITS, Cook's distance (cook.d), covariance ratio (cov.r), leave-one-out change in between-study variance (tau2.del), leave-one-out change in residual heterogeneity (QE.del), leverage (hat), and study weights (weight). Dashed lines mark reference thresholds; red points denote observations flagged as potentially influential/outlying. Panel (b) displays the same diagnostics after correcting for outliers by removing the flagged observations from panel (a). The stabilization of cov.r, tau2.del, QE.del, and weight profiles indicates that the overall effect estimate for species richness is robust to these studies, supporting the consistency of our main conclusions.

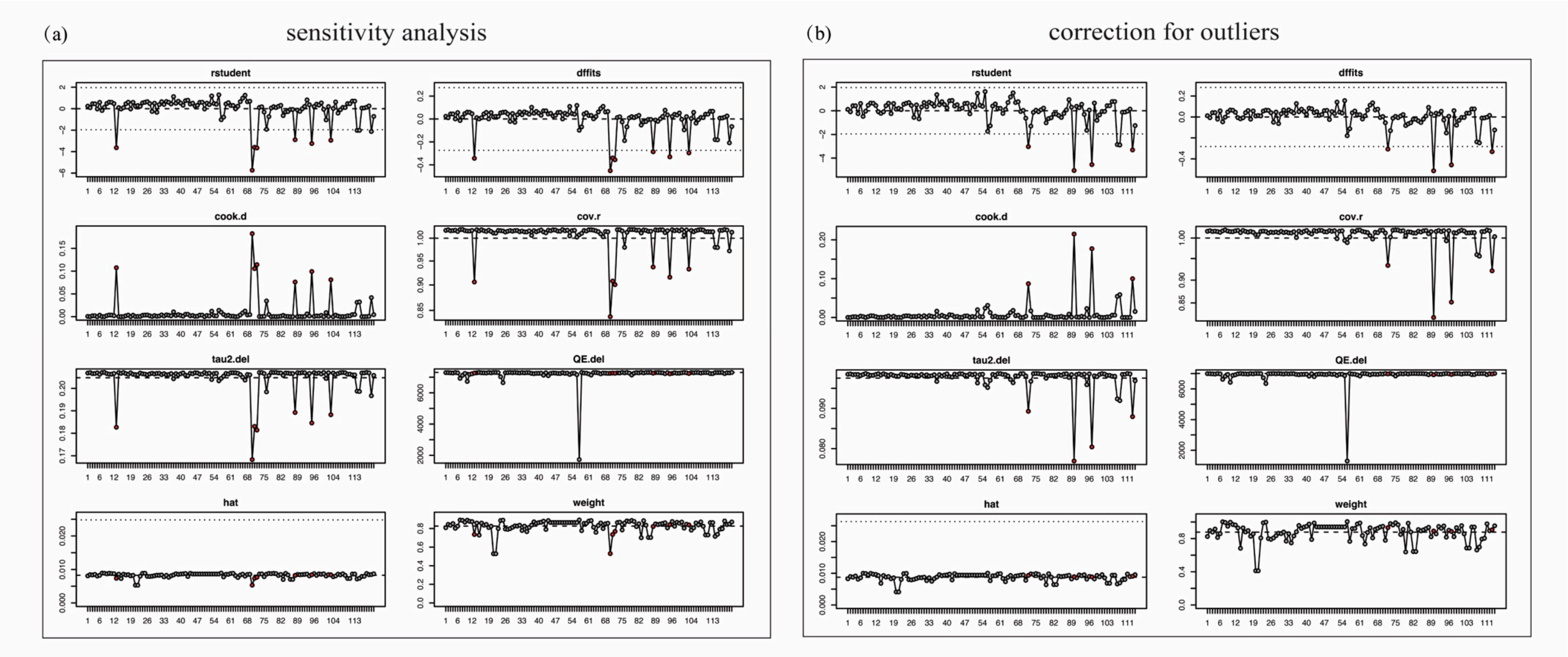

Figure S22. Sensitivity analysis for the meta-analysis of soil water content (SWC) responses to snow removal. Panel (a) shows influence diagnostics from a random-effects model: studentized residuals (rstudent), DFFITS, Cook's distance (cook.d), covariance ratio (cov.r), leave-one-out change in between-study variance (tau2.del), leave-one-out change in residual heterogeneity (QE.del), leverage (hat), and study weights (weight). Dashed lines mark reference thresholds; red points denote observations flagged as potentially influential/outlying. Panel (b) displays the same diagnostics after correcting for outliers by removing the flagged observations from panel (a). The stabilization of cov.r, tau2.del, QE.del, and weight profiles indicates that the overall effect estimate for SWC is robust to these studies, supporting the consistency of our main conclusions.

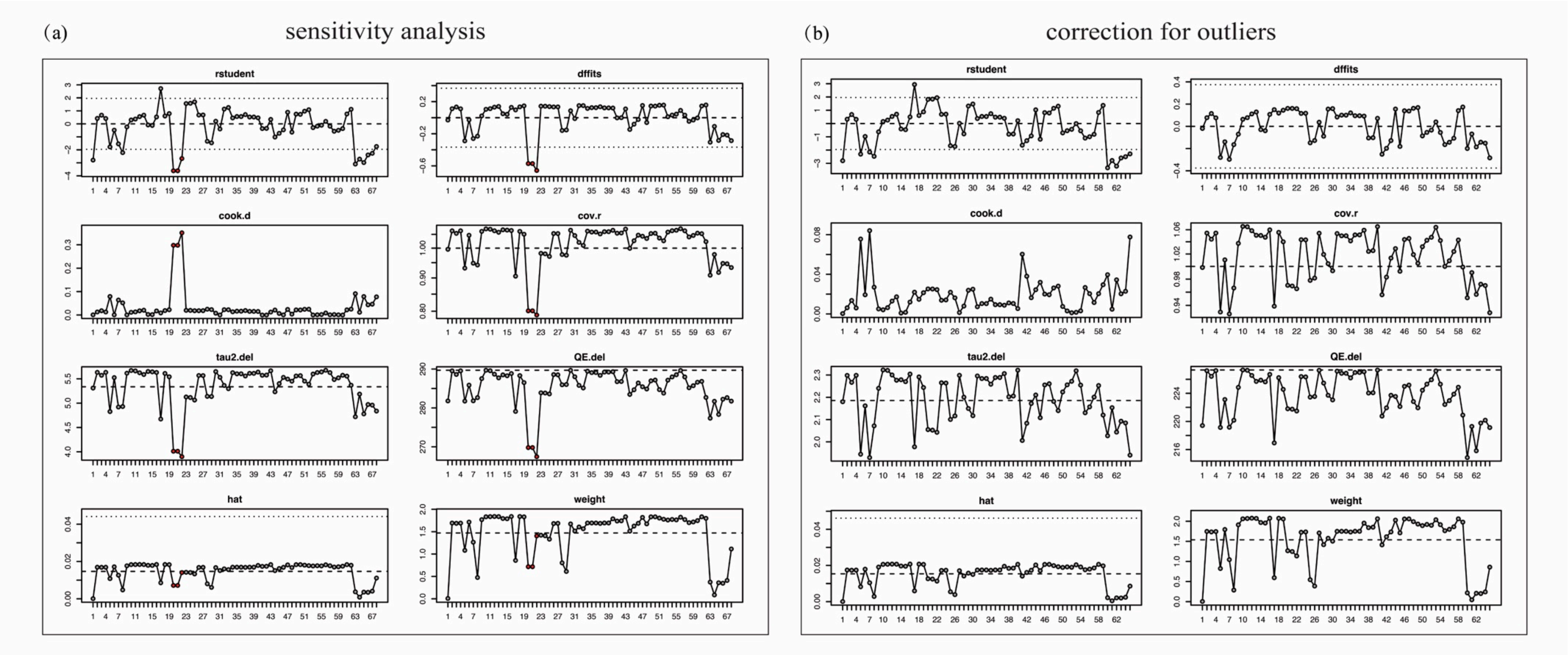

Figure S23. Sensitivity analysis for the meta-analysis of soil temperature (T) responses to snow removal. Panel (a) shows influence diagnostics from a random-effects model: studentized residuals (rstudent), DFFITS, Cook's distance (cook.d), covariance ratio (cov.r), leave-one-out change in between-study variance (tau2.del), leave-one-out change in residual heterogeneity (QE.del), leverage (hat), and study weights (weight). Dashed lines mark reference thresholds; red points denote observations flagged as potentially influential/outlying. Panel (b) displays the same diagnostics after correcting for outliers by removing the flagged observations from panel (a). The stabilization of cov.r, tau2.del, QE.del, and weight profiles indicates that the overall effect estimate for T is robust to these studies, supporting the consistency of our main conclusions.

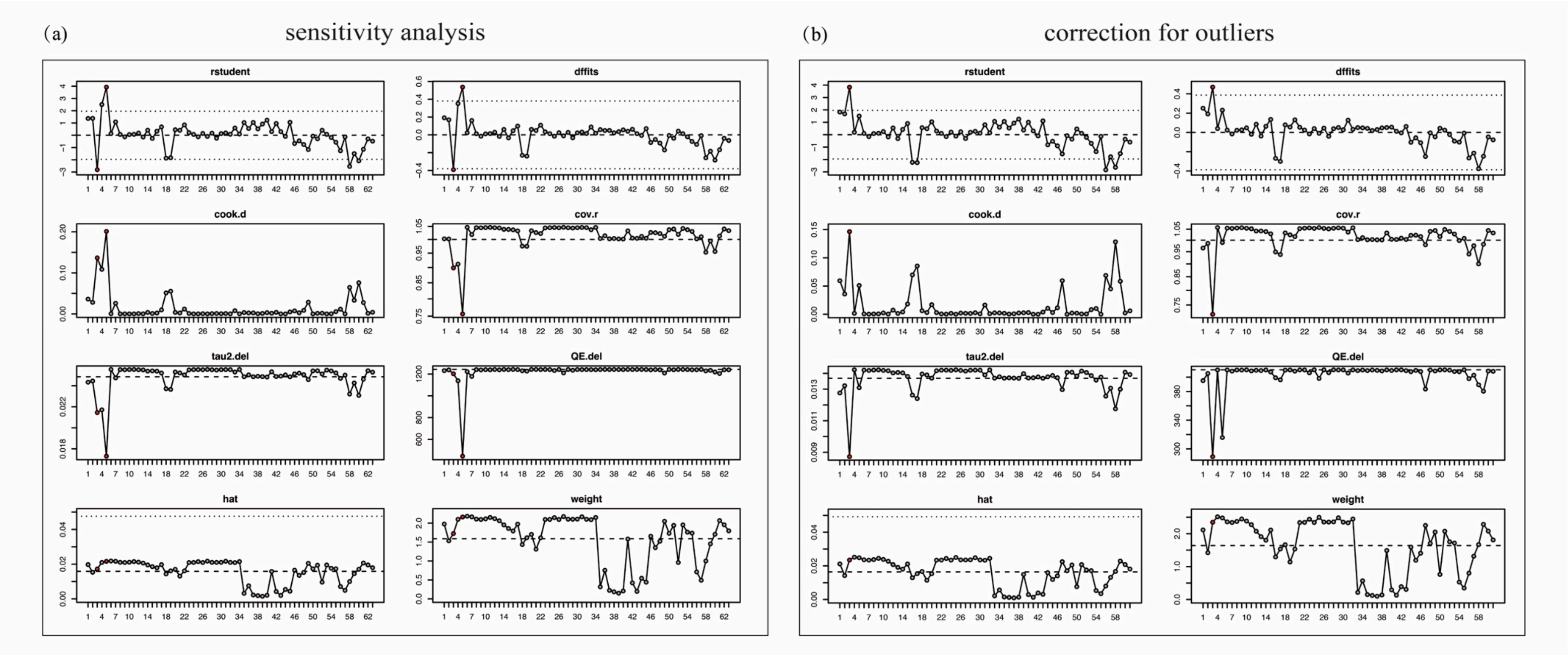

Figure S24. Sensitivity analysis for the meta-analysis of soil total nitrogen (TN) responses to snow removal. Panel (a) shows influence diagnostics from a random-effects model: studentized residuals (rstudent), DFFITS, Cook's distance (cook.d), covariance ratio (cov.r), leave-one-out change in between-study variance (tau2.del), leave-one-out change in residual heterogeneity (QE.del), leverage (hat), and study weights (weight). Dashed lines mark reference thresholds; red points denote observations flagged as potentially influential/outlying. Panel (b) displays the same diagnostics after correcting for outliers by removing the flagged observations from panel (a). The stabilization of cov.r, tau2.del, QE.del, and weight profiles indicates that the overall effect estimate for TN is robust to these studies, supporting the consistency of our main conclusions.

Table S1 Summary of effect sizes (*LnRR* ± 95% CI) for key ecosystem variables under snow addition treatment. Aboveground biomass, species richness, soil water content (SWC), total nitrogen (TN), and several microbial indicators showed positive or near-zero mean responses, whereas belowground productivity (BNPP) and some microbial metrics tended to decline or remain unchanged. Sample sizes (N) and heterogeneity statistics (*Q*df, *p*) are reported for each variable. Together, these results indicate that snow addition exerts heterogeneous but generally modest effects across ecosystem components, with the strongest responses observed in aboveground production and soil moisture.

name	estimate	se	ci.lb	ci.ub	N	Qdf	Q-pval
aboveground biomes	0.2583	0.1127	0.0375	0.4791	58	3378.224	< .0001
species richness	0.1352	0.0923	-0.0458	0.3162	30	145.0821	< .0001
underground biomes	0.2913	0.2519	-0.2023	0.785	5	5.6705	0.2251
community density	0.0003	0.0329	-0.0642	0.0647	9	5.6761	0.6835
ANPP	0.6014	0.352	-0.0885	1.2912	8	66.5302	< .0001
BNPP	-0.2715	0.1625	-0.59	0.047	6	25.3875	< .0001
Temperature	0.349	0.6394	-0.9041	1.6022	74	708.5521	< .0001
soil NH4	0.0079	0.0789	-0.1468	0.1627	72	792.2526	< .0001
soil NO3	0.0451	0.0968	-0.1446	0.2349	76	713.6335	< .0001
soil ph	0.0037	0.0047	-0.0055	0.013	88	344.4199	< .0001
soil SOC	0.0468	0.0298	-0.0116	0.1051	74	1213.6812	< .0001
soil SWC	0.0449	0.0542	-0.0613	0.1511	140	6908.1695	< .0001
soil TN	0.0136	0.0421	-0.069	0.0962	74	397.2183	< .0001
microorganism SMC	-0.0638	0.0899	-0.24	0.1124	75	5537.4182	< .0001
microorganism SMN	0.0315	0.066	-0.0978	0.1608	55	2194.9877	< .0001
microorganism Chao1	-0.0093	0.0361	-0.0801	0.0615	10	14.8791	0.0943

microorganism Observed species	0.0592	0.0338	-0.007	0.1254	6	0.2503	0.9985
microorganism OTU	-0.1388	0.183	-0.4975	0.2199	4	1.6686	0.6439
microorganism Shannon	-0.0027	0.0071	-0.0166	0.0113	10	13.3062	0.1492
microorganism Simpson	0.217	0.1401	-0.0575	0.4915	2	0.1296	0.7189

Table S2 Summary of effect sizes (*LnRR* ± 95% CI) for major ecosystem variables under snow removal treatment. Aboveground biomass, species richness, soil water content (SWC), total nitrogen (TN), and most microbial indicators showed negative or near-zero mean responses, whereas a few belowground or microbial metrics displayed weakly positive shifts. Sample sizes (N) and heterogeneity statistics (*Q*df, *p*) are provided for each variable. Collectively, these results suggest that snow removal produces heterogeneous but generally suppressive effects across ecosystem components, with the strongest decreases occurring in belowground productivity and soil moisture.

name	estimate	se	ci.lb	ci.ub	N	Qdf	Q-pval
aboveground biomes	-0.1852	0.1702	-0.5188	0.1484	38	14354.377	< .0001
species richness	-0.1044	0.0929	-0.2865	0.0777	17	42.4717	0.0003
underground biomes	0.1201	0.1932	-0.2584	0.4987	6	12.8391	0.0249
community density	-0.2694	0.2697	-0.7981	0.2593	12	44.7222	< .0001
ANPP	-0.2694	0.2697	-0.7981	0.2593	14	44.7222	< .0001
BNPP	-0.6158	0.0853	-0.7829	-0.4487	3	1.5104	0.4699
Temperature	-1.7981	1.0909	-3.9361	0.34	68	289.6987	< .0001
soil NH4	-0.0224	0.0412	-0.1032	0.0583	126	917.4119	< .0001
soil NO3	-0.0494	0.0535	-0.1543	0.0555	130	1562.7906	< .0001
soil ph	-0.003	0.0021	-0.0071	0.0011	91	248.5669	< .0001
soil SOC	-0.0064	0.0275	-0.0604	0.0476	89	965.886	< .0001

soil SWC	-0.2791	0.0785	-0.4329	-0.1252	121	7306.9531	< .0001
soil TN	0.0003	0.05	-0.0977	0.0983	63	1242.4634	< .0001
microorganism SMC	-0.0245	0.0492	-0.121	0.072	159	18329.671	< .0001
microorganism SMN	0.0229	0.0536	-0.0823	0.128	109	1501.2343	< .0001
microorganism Chao1	0.0155	0.0219	-0.0274	0.0584	19	57.2303	< .0001
microorganism Observed species	0.098	0.0476	0.0047	0.1912	5	3.2933	0.51
microorganism OTU	-0.0438	0.0893	-0.2189	0.1313	2	2.3934	0.1218
microorganism Shannon	-0.0098	0.0037	-0.0171	-0.0026	22	43.2104	0.003
microorganism Simpson	-0.0026	0.0084	-0.019	0.0138	13	27.2858	0.007
microorganism ACE	0.099	0.0202	0.0594	0.1386	3	0.3034	0.8592

Table S3 Summary of effect sizes (*LnRR* ± 95% CI) for soil variables at different depths under snow addition treatment. Snow addition generally increased soil water content (SWC), soil organic carbon (SOC), and total nitrogen (TN) across 0–5 cm, 5–15 cm and >15 cm layers, although the magnitude and direction of responses varied by depth and indicator. Ammonium (NH_4^+), nitrate (NO_3^-) and microbial metrics (SMC, SMN) showed heterogeneous responses, with some shifts reversing between shallow and deeper layers. Sample sizes (N) and heterogeneity statistics (Qdf, *p*) are reported for each variable. Together, these results indicate that snow addition exerts depth-dependent but generally positive effects on soil moisture and nutrient pools, while microbial responses remain variable.

**name**	**depth**	**estimate**	**se**	**ci.lb**	**ci.ub**	**N**	**Qdf**	**Q-pval**
SWC	0-5cm	0.1183	0.0547	0.0111	0.2255	106	1280.8169	< .0001
SWC	5-15cm	0.0512	0.0221	0.0079	0.0946	19	47.6099	0.0002
SWC	>15cm	0.1055	0.0147	0.0768	0.1343	9	19.2596	0.0135
NH4	0-5cm	0.0414	0.122	-0.1977	0.2805	39	288.7267	< .0001
NH4	5-15cm	-0.1135	0.0914	-0.2926	0.0656	25	468.5809	< .0001

NH4	>15cm	-0.0432	0.0681	-0.1768	0.0904	7	14.2795	0.0267
NO3	0-5cm	0.0849	0.1482	-0.2056	0.3753	40	331.7705	< .0001
NO3	5-15cm	-0.1533	0.1597	-0.4664	0.1597	25	332.1617	< .0001
NO3	>15cm	0.0424	0.0753	-0.1051	0.1899	10	42.6825	< .0001
SMC	0-5cm	-0.0133	0.0653	-0.1413	0.1146	32	441.5913	< .0001
SMC	5-15cm	0.0368	0.0458	-0.0529	0.1265	24	93.7271	< .0001
SMC	>15cm	0.0979	0.1325	-0.1618	0.3576	1	0	1
SMN	0-5cm	-0.0436	0.0958	-0.2314	0.1442	33	1939.541	< .0001
SMN	5-15cm	0.134	0.0659	0.0048	0.2632	22	125.6687	< .0001
SOC	0-5cm	0.0444	0.0369	-0.028	0.1168	35	318.072	< .0001
SOC	5-15cm	0.0297	0.0353	-0.0396	0.0989	24	329.5501	< .0001
SOC	>15cm	0.2203	0.0813	0.0609	0.3797	9	197.9957	< .0001
TN	0-5cm	0.0167	0.0389	-0.0595	0.093	37	67.3423	0.0012
TN	5-15cm	0.0167	0.0823	-0.1445	0.178	34	318.0168	< .0001
Temperature	0-5cm	0.1734	0.5354	-0.8761	1.2228	57	660.547	< .0001
Temperature	5-15cm	0.7268	1.6474	-2.5022	3.9557	13	35.753	0.0004
Temperature	>15cm	-0.7197	1.3591	-3.3834	1.944	4	9.1354	0.0275
PH	0-5cm	0.0055	0.0025	0.0006	0.0103	40	41.8388	0.3486
PH	5-15cm	0.003	0.0117	-0.02	0.0259	29	233.9945	< .0001
PH	>15cm	0.0063	0.0068	-0.0072	0.0197	14	45.9649	< .0001

Table S4 Summary of effect sizes (*LnRR* ± 95% CI) for soil variables at different depths under snow removal treatment. Snow removal generally

decreased soil water content (SWC), soil organic carbon (SOC) and total nitrogen (TN) across 0–5 cm and 5–15 cm layers, while effects in deeper layers (>15 cm) were weak or inconsistent. Ammonium (NH_4^+), nitrate (NO_3^-) and microbial metrics (SMC, SMN) also showed depth-dependent and heterogeneous responses, with some metrics switching direction between shallow and deeper soils. Sample sizes (N) and heterogeneity statistics (*Q*df, *p*) are reported for each variable. Together, these results indicate that snow removal exerts depth-specific but generally suppressive effects on soil moisture and nutrient pools, whereas microbial responses remain variable across depths.

name	depth	estimate	se	ci.lb	ci.ub	N	Qdf	Q-pval
SWC	0-5cm	-0.3273	0.1129	-0.5487	-0.106	80	1380.61	< .0001
SWC	5-15cm	-0.1423	0.1132	-0.3642	0.0795	22	2348.5731	< .0001
SWC	>15cm	0.0212	0.0207	-0.0193	0.0617	2	0.001	0.975
NH4	0-5cm	-0.0674	0.0639	-0.1927	0.0579	65	487.43	< .0001
NH4	5-15cm	0.0135	0.0515	-0.0873	0.1144	55	332.8243	< .0001
NH4	>15cm	0.1702	0.1338	-0.0921	0.4325	3	1.935	0.38
NO3	0-5cm	-0.1618	0.085	-0.3284	0.0048	66	977.2061	< .0001
NO3	5-15cm	0.0402	0.047	-0.0519	0.1323	56	281.6643	< .0001
NO3	>15cm	-0.0482	0.1284	-0.3	0.2035	3	3.746	0.1537
SMC	0-5cm	0.0028	0.059	-0.1129	0.1186	53	1924.2857	< .0001
SMC	5-15cm	-0.0449	0.0993	-0.2395	0.1498	89	13337.1383	< .0001
SMC	>15cm	0.0369	0.1225	-0.2033	0.2771	15	2344.4625	< .0001
SMN	0-5cm	0.0093	0.114	-0.2141	0.2327	45	1067.689	< .0001
SMN	5-15cm	0.0615	0.0557	-0.0477	0.1706	63	348.4459	< .0001
SMN	>15cm	0.2375	0.1408	-0.0385	0.5135	3	1.8625	0.3941
SOC	0-5cm	-0.0685	0.0448	-0.1563	0.0193	35	240.0965	< .0001

SOC	5-15cm	0.0568	0.0409	-0.0234	0.137	42	344.3967	< .0001
SOC	>15cm	-0.0988	0.065	-0.2263	0.0286	1	0	1
TN	0-5cm	-0.0578	0.0521	-0.1598	0.0443	38	140.3316	< .0001
TN	5-15cm	0.0404	0.1112	-0.1775	0.2584	18	50.6327	< .0001
TN	>15cm	0.0347	0.0453	-0.0542	0.1236	3	1.9285	0.3813
Temperature	0-5cm	-3.7555	2.5697	-8.7921	1.281	40	175.6678	< .0001
Temperature	5-15cm	-2.0226	1.0131	-4.0083	-0.0369	26	110.9602	< .0001
Temperature	>15cm	0.4298	0.7172	-0.9759	1.8355	2	0.0009	0.9756
PH	0-5cm	-0.0012	0.0025	-0.0061	0.0038	48	134.197	< .0001
PH	5-15cm	-0.001	0.0067	-0.0142	0.0122	25	71.0858	< .0001
PH	>15cm	-0.0052	0.0039	-0.0127	0.0024	9	10.6553	0.222
